# The spectrum of chromosomal translocations in the Arab world: ethnic-specific chromosomal translocations and their relevance to diseases

**DOI:** 10.1007/s00412-022-00775-2

**Published:** 2022-07-30

**Authors:** Hadeel T. Zedan, Fatma H. Ali, Hatem Zayed

**Affiliations:** 1 Biomedical Research Center, Qatar University, P.O. Box 2713, Doha, Qatar; 2 Department of Biomedical Science, College of Health Sciences, Member of QU Health, Qatar University, P.O. Box 2713, Doha, Qatar

**Keywords:** Chromosomal translocations, Arab countries, Genotype–phenotype correlations, Cancer

## Abstract

Chromosomal translocations (CTs) are the most common type of structural chromosomal abnormalities in humans. CTs have been reported in several studies in the Arab world, but the frequency and spectrum of these translocations are not well characterized. The aim of this study is to conduct a systematic review to estimate the frequency and spectrum of CTs in the 22 Arab countries. Four literature databases were searched: PubMed, Science Direct, Scopus, and Web of Science, from the time of inception until July 2021. A combination of broad search terms was used to collect all possible CTs reported in the Arab world. In addition to the literature databases, all captured CTs were searched in three chromosomal rearrangement databases (Mitelman Database, CytoD 1.0 Database, and the Atlas of Genetics and Cytogenetics in Oncology and Hematology), along with PubMed and Google Scholar, to check whether the CTs are unique to the Arabs or shared between Arabs and non-Arabs. A total of 9,053 titles and abstracts were screened, of which 168 studies met our inclusion criteria, and 378 CTs were identified in 15 Arab countries, of which 57 CTs were unique to Arab patients. Approximately 89% of the identified CTs involved autosomal chromosomes. Three CTs, t(9;22), t(13;14), and t(14;18), showed the highest frequency, which were associated with hematological malignancies, recurrent pregnancy loss, and follicular lymphoma, respectively. Complex CTs were commonly reported among Arabs, with a total of 44 CTs, of which 12 were unique to Arabs. This is the first study to focus on the spectrum of CTs in the Arab world and compressively map the ethnic-specific CTs relevant to cancer. It seems that there is a distinctive genotype of Arabs with CTs, of which some manifested with unique clinical phenotypes. Although ethnic-specific CTs are highly relevant to disease mechanism, they are understudied and need to be thoroughly addressed.

**Supplementary Information:**

The online version contains supplementary material available at 10.1007/s00412-022-00775-2.

## Introduction

Chromosomal translocations (CTs) are genetic abnormalities that involve an exchange of segments between chromosomes, leading to unusual structural chromosomal rearrangements (Roukos and Misteli 2014). The consequences associated with CTs depend on the location of the breaks, which can lead to fusion of genes, gene disruption, or gene dysregulation (Wilch and Morton 2018). CTs are the most common type of structural chromosomal abnormalities found in humans and are classified into two main types, reciprocal and Robertsonian translocations (Vasilevska et al. 2013). Reciprocal translocation involves an exchange of segments between two non-homologous chromosomes. In contrast, Robertsonian translocations usually involve acrocentric chromosomes, in which the entire chromosome attaches to another chromosome at the centromere (Wilch and Morton 2018). Both types of translocations can be presented in balanced and unbalanced states (Roukos and Misteli 2014; Vasilevska et al. 2013).

Balanced translocations are usually not associated with phenotypic consequences and may pass undetected through generations (Wilch and Morton 2018). Although the estimates vary, balanced reciprocal translocations occur in about one per 300–500 individuals, whereas balanced Robertsonian translocations are more frequent and occur in about one per 100 individuals (Wilch and Morton 2018; Priya et al. 2018). Moreover, balanced translocations have been associated with recurrent pregnancy loss. For example, among 2–5% of couples suffering from frequent miscarriages, one of the partners was found to be a carrier of a balanced translocation (Dutta et al. 2011; Sheth et al. 2013). On the other hand, unbalanced translocations are less common. Still, they could lead to significant clinical anomalies such as monosomy and trisomy, accounting for around 1% of developmental delay and intellectual disability cases (Weckselblatt et al. 2015). Further, unbalanced translocations detected in affected children could arise de novo or may be inherited from a parent carrying a balanced translocation (Weckselblatt et al. 2015).

CTs are clinically relevant as they play key roles in several human cancers and non-cancerous diseases with a de novo frequency of one in 2000 (Roukos and Misteli 2014). Chromosomal aberrations have long been considered a characteristic feature of neoplasia, where acquired CTs have been reported in more than 50,000 cases of different cancer types (Rowley 2001). In addition, there is compelling evidence that CTs play a critical role in the initial pathogenesis events of about 20% of cancers, although the exact mechanism is not fully understood (Forabosco et al. 2009). CTs are also used as decisive diagnostic indicators for detecting several clinical syndromes using molecular cytogenetic techniques (Mitelman et al. 2007). The development of fluorescence in situ hybridization (FISH), multicolor FISH, and comparative genomic hybridization (CGH) have enabled the specific detection of unique sequences, chromosomal regions, and entire chromosomes for the identification of numerous chromosomal abnormalities implicated in oncogenesis (Nowakowska and Bocian 2004).

Although the spectrum of variants causing single-gene disorders (Al-Sadeq et al. 2019; Doss et al. 2016; Khan et al. 2021; Mosaeilhy et al. 2017; Zaki et al. 2017; Zayed 2015a, 2015b, 2015c) and associated with multifactorial diseases (Abuhendi et al. 2019; Al-Thani et al. 2021; Alhababi and Zayed 2018; Jemmeih et al. 2022; Younes et al. 2020; Younes et al. 2021; Younes and Zayed 2019; Zayed 2016a; Alsamman and H., Zayed, H., 2022) were reviewed in the Arab countries, the spectrum and frequency of CTs among Arab countries and their relevance to diseases have not been reported yet. Therefore, this systematic review aimed to explore the spectrum of CTs in the Arab world and their association with diseases.

## Materials and methods

### Search strategy

Four databases were searched (PubMed, Science Direct, Scopus, and Web of Science) for all articles published in English from the time of inception until July 2021. Search terms were broad to capture all conducted studies; this includes “Chromosomal translocation,” in combination with each of the 22 Arab countries, for example, “Iraq AND chromosomal translocation.” In addition, relevant articles were screened for both titles and abstracts for their eligibility.

### Study selection

The studies included in this review were selected based on the following inclusion criteria: (i) published in peer-reviewed journals, (ii) conducted on Arab patients residing in Arab countries, (iii) contained data on Arab patients diagnosed with any CTs, (iv) contained data about the frequency of Arab patients with CTs, and (v) Arabs residing in only Arab countries. Articles were excluded if they did not meet the inclusion criteria. All citations were exported to Endnote version X9, and duplicated articles were removed.

### Data extraction and analysis

The collected data was reviewed twice by HTZ and FTA; another layer of revision was done by the senior author HZ to ensure that the data had been captured correctly. The eligible articles were fully screened, and the data related to the CTs were extracted, including disease, country, type of CTs, patients’ karyotype, age, number of patients screened, clinical phenotypes, method of CTs detection, association with other genetic abnormalities, and presence of consanguinity. To gain a better understanding of the ethnic distributions of the captured CTs, and identify whether they are unique to Arab populations or shared with other ethnic groups, in addition to literature search, all captured CTs were searched in the following databases: Mitelman Database (https://mitelmandatabase.isb-cgc.org/search_menu), CytoD 1.0 Database (http://www.changbioscience.com/cytogenetics/cyto1.pl), the Atlas of Genetics and Cytogenetics in Oncology and Hematology (http://atlasgeneticsoncology.org/), PubMed, and Google Scholar.

## Results

### Search findings

**Fig. 1:**
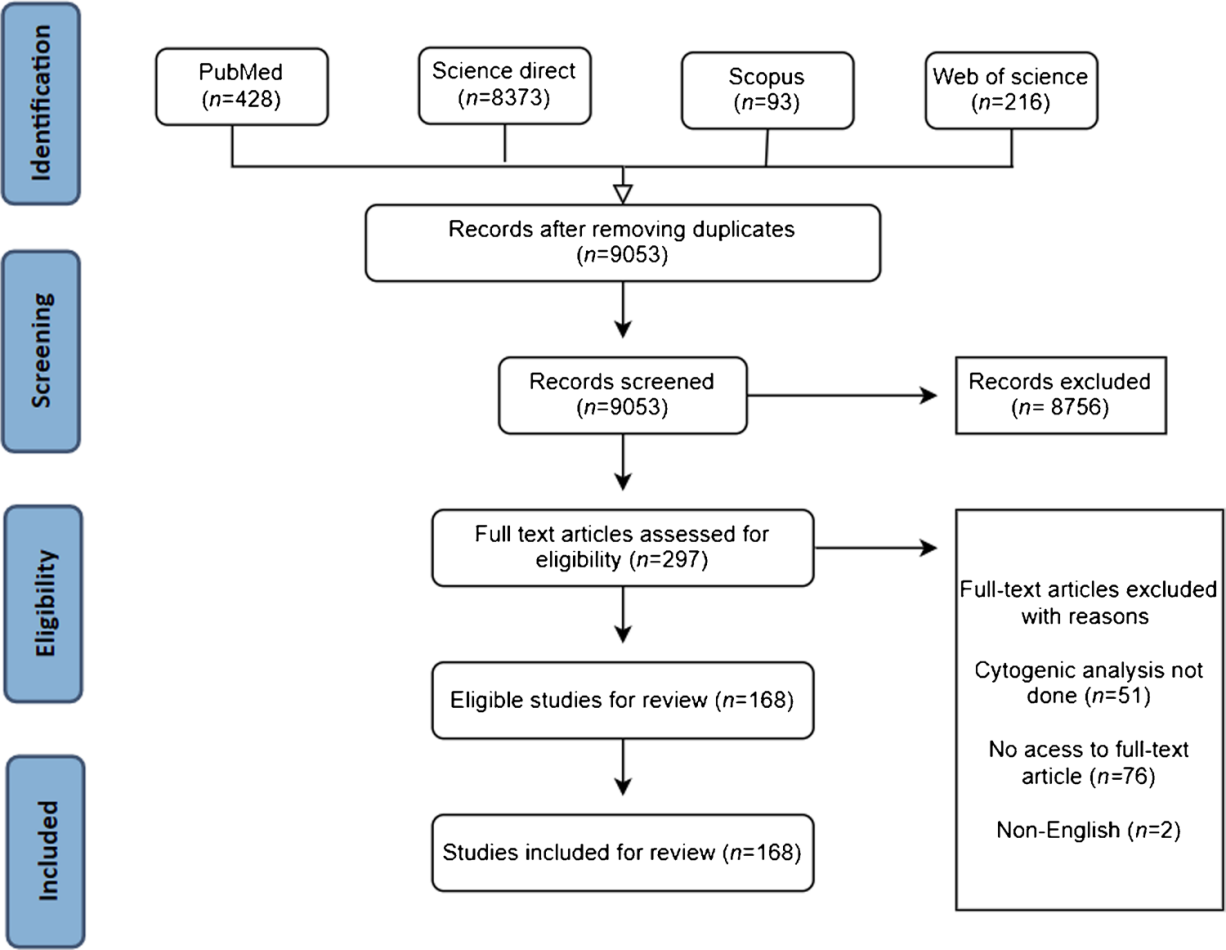
Flow diagram of the selected articles

The search strategy identified 9,110 citations, of which 9,053 remained after removing duplicates. A total of 8,756 citations were irrelevant and therefore excluded. After the abstract screening, 297 citations were thoroughly screened for the inclusion criteria as described in the Methods section. Of these, 168 studies were eligible and included in our systematic analysis (Fig. 1). All reported CTs in the analysis were checked through several chromosomal rearrangement databases, as indicated in the “Methods” section, to identify their clinical significance and determine whether they are unique to the Arab populations or not.

### The frequency of CTs among Arabs and their clinical findings

**Fig. 2:**
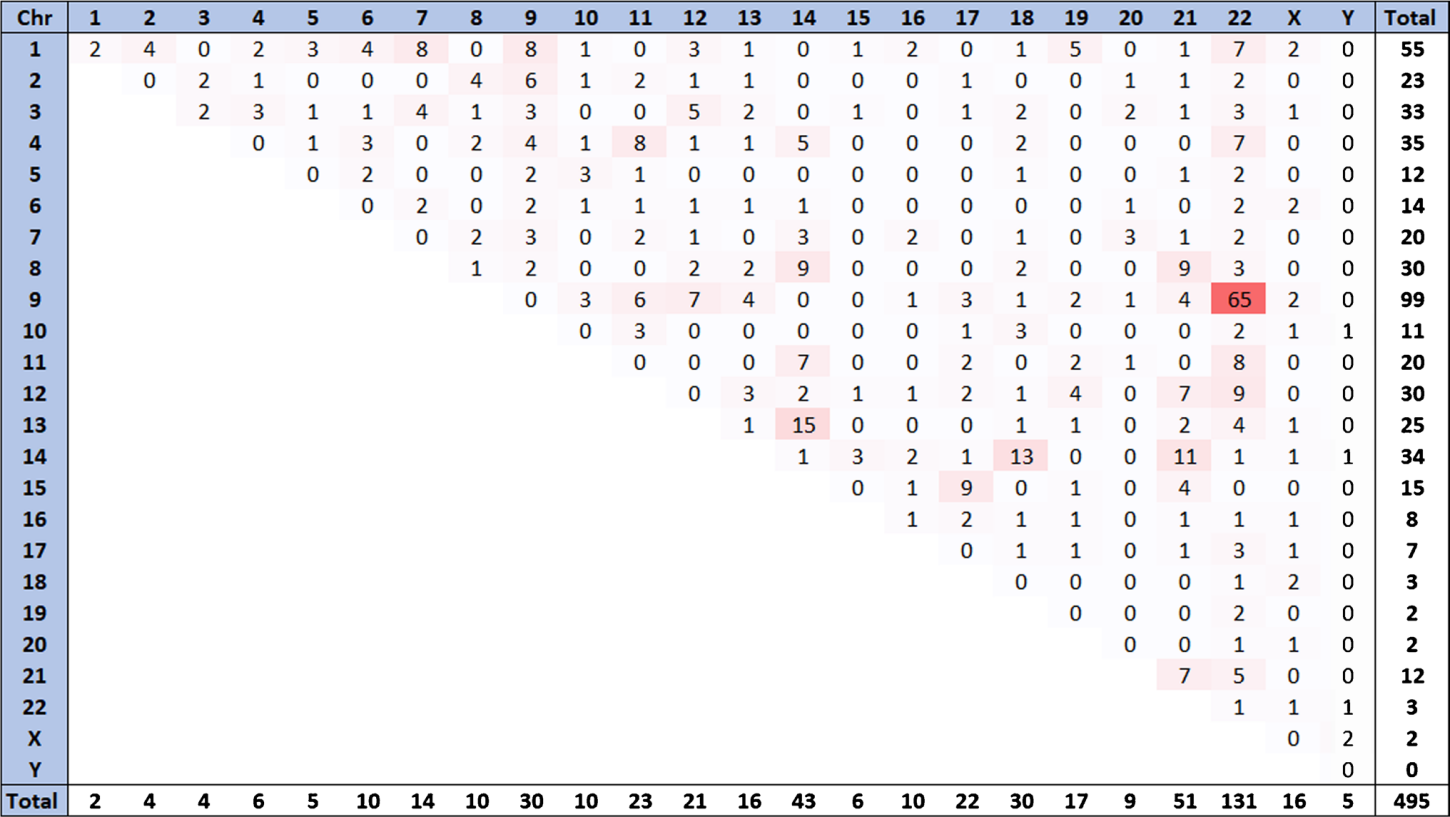
Distribution of the different combinations of the chromosomes involved in chromosomal translocations in the Arab Countries. Chr: chromosome

The CTs and their clinical significance captured in our study are summarized in Table 1, Table 2, and Table S1. Our strategy identified Arab individuals with CTs in 15 out of the 22 Arab countries. No studies were captured in Bahrain, Comoros, Djibouti, Iraq, Mauritania, Somalia, and Yemen. The highest number of CTs was reported in Egypt (*n* = 93), followed by Tunisia (*n* = 69), Morocco (*n* = 46), Syria (*n* = 36), Saudi Arabia (*n* = 31), Oman (*n* = 29), Qatar (*n* = 22), Lebanon (*n* = 16), Jordan (*n* = 12), Kuwait (*n* = 11), Algeria (*n* = 6), Sudan (*n* = 3), and UAE (*n* = 2), while only one CT was reported in each of Libya and Palestine. A total of 378 CTs were reported in individuals belonging to the 15 Arab countries (Table S1), of which 57 CTs were unique to Arabs (i.e., reported in Arabs and not reported in any other ethnic groups) (Table 1), and 321 CTs were shared with other ethnic groups (Table S1). Of the 378 CTs, 190 (50.3%) were reciprocal including 27 *de novo* and 22 familial CTs, whereas 40 (10.6%) were Robertsonian including 12 *de novo* and eight familial CTs. Further, CTs frequency among Arabs showed males preponderance. All autosomal and sex chromosomes were involved in the captured CTs (Fig. 2). The majority (89.4%) of the CTs involved the autosomal chromosomes. Chromosomes 22, 9, 1, 21, 14, 3, 18, 8, and 12 were the most frequently involved chromosomes, while chromosome Y was the least involved. Three translocations showed the highest frequency among Arabs: (i) t(9;22) in Egypt, Jordan, Kuwait, Lebanon, Morocco, Oman, Qatar, Saudi Arabia, Syria, and Tunisia; (ii) t(13;14) in Egypt, Morocco, Oman, Qatar, Saudi Arabia, and Tunisia; and (iii) t(14;18) in Egypt, Jordan, Lebanon, Saudi Arabia, and Tunisia. There were only 16 autosome-sex CTs: t(X;1), t(X;3), t(X;6), t(X;9), t(X;10), t(X;13), t(X;14), t(X;16), t(X;17), t(X;18), t(X;20), t(X;9;22), t(X;Y), t(Y;10), t(Y;14), and t(Y;22), which were reported in Egypt, Jordan, Morocco, Oman, Qatar, Syria, and Tunisia (Fig. 2).

**Table 1:** The unique chromosomal translocations among Arab countries

Disease	Arabic country	Translocation’s type	Karyotype	Familial/de novo	Age/ sex	No. of patients/No. screened	Clinical phenotype	Primary mutation/associated with other abnormalities	Consanguinity	Reference
RPL with birth deformities	Egypt	Rec^†^ Rec^†^ Rec^†^	t(3;8)(p25;p11) t(4;6)(p24;q25) t(7;21)(p11;p11)	De novo **-** **-**	30y/M 22y/F 22y/F	1/- 1/- 1/-	Repeated abortions, stillbirth, fetal malformation, and birth of mentally handicapped children	-	-	Gaboon et al. 2015)
RPL		Rec Rec Rec Rec	t(1;15)(p35;q15) t(3;15)(p23;q26.2) t(3;7)(p26;p15) t(4;6)(q25;q26)	Fam	23-50y/ 7F, 5 M	12/224	Recurrent abortions and the birth of dysmorphic/mentally handicapped infants	Primary	Consanguineous couple involving translocation in chromosomes 11 and 12	Elhady et al. 2020)
Therapy-related acute myeloid leukemias		Rec	t(v;11q23)	De novo	Median: 37y/ 46 M, 28F	6/120	Poor topoisomerase II inhibitor treatment outcome	Primary	-	Mosad et al. 2012)
Down syndrome		Rec	t(4;21)(q25;q22)	Mat	8y/F	1/1	Severe growth retardation, microcephaly, hearing impairment, and specific facies	Associated with partial trisomy 4q25-qter and 21(pter-q22)	-	El-Ruby et al. 2007)
RPL		- -	t(16;X)(q24;q23) t(3;22)(q11;p11)	-	39 yr/M 6.5 yr/M 38 yr/M	2/73 1/73	Recurrent miscarriage	-	-	El-Dahtory 2011)
Congenital anomalies		-	t(X:13)(p22.2:q12)	-	-	-	Physical disabilities, stillbirths, and neonatal deaths	-	Consanguinity reported in 43% of couples	AbouEl-Ella et al. 2018)
AML	Jordan	Rec	t(15;16;17;19)	-	58y/F	1/1	Acute promyelocytic leukemia (AML-M3)	Primary complex translocation	-	Kamal et al. 1996a)
Unbalanced chromosomal rearrangement		-	t(5;10)(q35;q25)	Pat	6 m/F	5/-	Developmental delay, hypotonia, supernumerary nipples, and distinct craniofacial features	Associated with der(10)	No	Masri et al. 2014)
Constitutional jumping translocations	Lebanon	-	t(8;18)(q24.3,p11.2)	-	26y/M	1/1	Partial hypogonadism	Associated with ring chromosome 18	-	Zahed et al. 2004)
Spontaneous RPL	Kuwait	-^†^	t(7:11) (p10:q10)	De novo	37y/F	1/1	High-order miscarriage	Associated with other etiological factors	No	Diejomaoh et al. 2015)
CML		Rec	t(9;22;12)(q34;q11;p11)	-	26y/M	1/1	Similar to CML clinical features	Primary	-	Zámecˇníkova et al. 2012)
CML		-	t(9;22;7;1)(q34;q11;q22;p13)	-	64y/M	1/1	Similar prognosis to those with classical Ph translocations	Associated with tyrosine kinase inhibitor therapy	-	Adriana and Al Bahar 2012a)
Intellectual disability	Morocco	Rec Rec	t(2;17)(q12;q23) t(21;21)(p11;p11)	-	-/8 M, 6F	14/1200	Non-syndromic intellectual disability	Primary	-	Belkady et al. 2018)
Spontaneous RPL		Rec Rec Rec Rec	t(2;11)(p14;q13) t(2;8)(p22;p22) t(3;13)(q24;q34) t(3;18)(q28;q22)	-	-	4/1254	Recurrent spontaneous miscarriage	Primary	-	Elkarhat et al. 2019) (Elkarhat et al. 2019)
		Rob Rob	t(21;21)(p11;p11) t(13;13)(q10;q10)	-	-	2/1254				
ALL	Oman	-	t(5;11)(q13;p12)	-	0.7y/M	93/120	Pre-B ALL	-	-	Goud et al. 2015)
RPL		Rec Rec	t(1;12)(q32;q24) t(1;5)(qter;p14)	-	29y/M 29y/M	18/760	Miscarriage occurrence of at least two times	Primary	-	Goud et al. 2009)
Azoospermia and severe oligozoospermia	Qatar	Rec Rec	t(2;9)(p21;p22) t(Y;10)(q11.2; q24)	-	-/M	49/511	Azoospermia, severe oligozoospermia and infertility in men	-	-	Arafa et al. 2018)
		Rob	t(15;21)(q10;q10)	-	-/M					
RPL	Saudi Arabia	Rec Rec	t(3;4;13;6)(q25;q32;q31;q22) t(3;7)(p23;p22)	-	-/M 33y/F	1/171 1/171	High average of pregnancy failures	Associated with factor V Leiden and prothrombin A20210G allelic polymorphisms	33% of couples had family history of consanguineous marriages	Turki et al. 2016a)
Unbalanced inherited translocation		Rec	t(1;7)(1q42.3q44,7q36.1q36.3)	Mat	-	1/5	Neurological phenotype and brain malformation	Primary	Yes	AlMajhad et al. 2017)
Type 1 diabetes		Rec	t(4;18)(q34.2;p11.2)	Pat	21y/M	3/7	Microcephaly, ectodermal dysplasia, hepatosplenomegaly	Associated with -18, + der (18)	Yes	Cherian 2012)
Pure erythroid leukemia		-	t(8;9)(p11.2;q12)	-	48y/M	1/1	Pancytopenia and circulating erythroblast in peripheral blood	Associated with del(5q) and del(7q)	-	Aljabry 2015)
CHARGE syndrome		-	t(4;8)(q34;q22.1)	De novo	2 m/M	1/1	choanal atresia, facial dysmorphism, cardiovascular malformations, and developmental delay	Primary	No	Khalifa et al. 2011)
Intellectual disability		-^‡^	t(13;18)(q34,q23)	De novo	13y/M 11y/M	2/2	Intellectual disability, obesity, dysmorphic features, speech delay, and seizure	Associated with 13q34 microdeletion, 18q23 microduplication, and 6q25 deletion	-	Alhashem, et al. 2020)
APL	Syria	Rec	t(1;2)(q42 ~ 43;q11.2 ~ 12)	De novo	46y/F	1/1	Multiple sclerosis, fatigue, loss of weight, fever, and an elevated WBC count	Two associated translocations	-	Wafa, et al. 2016)
CML		Rec	t(9;10;22)(q34;p11.2;q11.2)	-	42y/M	1/1	Imatinib mesylate-resistant CML	Primary	-	Al-Achkar, et al. 2013a)
CML		Rec Rec	t(9;22)(q34;q11) t(16;17)(p13.3;17q21 to 17qter)	-	30y/M	1/1	CML with complex secondary chromosomal changes, treatable with imatinib	Associated with partial trisomy of 17q21 to 17qter and trisomy 9	-	Al Achkar, et al. 2010)
CML		Rec Rec	t(12;19)(p11.2;q13.3) t(9;12;19;22)	-	25y/F	1/1	Similar to CML clinical features	Complex with trisomy 8 and a derivative chromosome 12	-	Al Achkar et al. 2010a)
CML		Rec	t(5;9;22)(p15.1; q34; q11.2)	-	-	1/1	-	Complex	-	Al-Achkar et al. 2007a)
CML		-	t(1;4;5;9;22)(q42;p14;q31;q34;q11.2)	*-*	45y/F	1/1	CML in chronic phase	Complex	-	Al Achkar et al. 2009a)
Follicular lymphoma and B-cell lymphoblastic leukemia		- -	t(3;20)(q26.2;q12) t(X;9)(p21.3;q22.3)	De novo	38/F	1/1	Adult FL grade 2 transformed to B-ALL	Complex	-	Wafa et al. 2016)
RPL	Tunisia	Rec	t(4;10)(q28;q25)	-	-/F	1/326	Recurrent miscarriage	Primary	-	Ayed et al. 2017a)
Infertility		Rec	t(9;13)(q33;q22)	-	Mean: 36.8y/M	2/6	Reproductive failure (recurrent miscarriage, infertility problem)	Primary	-	Hajlaoui et al. 2018b)
Mental retardation and spina bifida		Rec	t(2;3)(q35;p26.2)	De novo	6y/F	1/1	Mental retardation, mild growth, congenital malformation, and facial anomalies	Associated with partial trisomy 2q35 and partial monosomy 3p26	No	Abdallah et al. 2011)
CML with variant Ph-rearrangements		Rec Rec Rec Rec Rec Rec	t(1;1;2;9;12;13;22)(q24;q31;p21;q34;q11.2) t(1;1;9;22)(p34;q42;q34;q11.2) t(4;9;22)(q13;q34;q11.2) t(4;9;22)(q27;q34;q11.2) t(4;9;22)(q34;q34;q11.2) t(9;12;22)(q34;p13;q11.2)	-	-	1/336 1/336 1/336 1/336 1/336 1/336	Similar to CML clinical features	Associated with deletions	-	Bennour et al. 2009a)
AML		-	t(X;10)(pl0;pl0)	-	86y/M 27y/M	2/-	AML with poor prognosis due to systemic candidiasis and relapse	Primary associated with other abnormalities	-	Bennour, et al. 2010)

**Abbreviations**: *Rec*, reciprocal; *Rob*, Robertsonian; *y*, years; *m*, months; *d*, days; *fam*, familial; *pat*, paternal; *mat*, maternal; *ALL*, acute lymphoblastic leukemia; *AML*, acute lymphocytic leukemia; *RPL*, recurrent pregnancy loss; *CML*, chronic myeloid leukemia; *CHARGE syndrome*, coloboma, heart defects, atresia choanae, growth retardation, genital abnormalities, and ear abnormalities; *APL*, acute promyelocytic leukemia

†balanced translocation, ‡unbalanced translocation

**Table 2:** The complex chromosomal translocations (CTs) reported among Arab countries

Disease	Arabic country	Translocation’s type	Karyotype	Familial/de novo	Age/ sex	No. of patients/No. screened	Clinical phenotype	Primary mutation/associated with other abnormalities	Consanguinity	Reference
Burkitt’s lymphoma	Algeria	Rec^**^	t(2;8;9)	De novo	9y/M	1/22	Jaw and abdominal tumors, facial asymmetry, enlarged lymph nodes, and abdominal masses	A three-way recombination with translocation and insertion	-	Philip et al. 1981)
AML	Jordan	Rec^*^	t(15;16;17;19)	-	58y/F	1/1	Acute promyelocytic leukemia (AML-M3)	Primary complex translocation	-	Kamal et al. 1996a)
AML	Lebanon	-^**^	t(8;12;21)(q22;p12 approximately p13;q22)	-	32y/M	1/1	AML (FAB- M2)	Associated with chromosomal abnormalities (loss of Y ch. and trisomy 8q22)	-	Farra et al. 2004)
CML	Kuwait	Rec^**^	t(9;22;12)(q34;q11;p11)	-	26y/M	1/1	Similar to CML clinical features	Primary	-	Zámecˇníkova et al. 2012)
CML		-^*^	t(9;22;7;1)(q34;q11;q22;p13)	-	64y/M	1/1	Similar prognosis to those with classical Ph translocations	Associated with tyrosine kinase inhibitor therapy	-	Adriana and Al Bahar 2012a)
CML	Morocco	-^**^	t(9;18;22)(q34;p11;q11)	-	29y/M	1/1	Similar to CML clinical features	Associated with der(18)	-	Andaloussi and Bilhou-Nabera 2007)
Intellectual disability		Rec^**^	t(1;6;7)(p21;q16;p21)	-	-/8 M, 6F	14/1200	Non-syndromic intellectual disability	Primary	-	Belkady et al. 2018)
AML	Oman	-^**^	t(8;13;21)(q22;q14;q22)	-	33y/F	1/1	AML-FAB M2	Primary	-	Udayakumar et al. 2008)
RPL	Saudi Arabia	Rec^*^	t(3;4;13;6)(q25;q32;q31;q22)	-	-/M	1/171	High average of pregnancy failures	Associated with factor V Leiden and prothrombin A20210G allelic polymorphisms	33% of couples had family history of consanguineous marriages	Turki et al. 2016a)
CML	Syria	Rec^**^	t(9;11;20;22)(q34;p11.2;q11.21;q11)	-	55y/F	1/1	No symptoms were observed, but the patient was lost during follow-up	Primary	-	Al-Achkar et al. 2013)
CML		Rec^*^	t(9;10;22)(q34;p11.2;q11.2)	-	42y/M	1/1	Imatinib mesylate-resistant CML	Primary	-	Al-Achkar, et al. 2013a)
CML		-^**^	t(1;2;9;22)(p32;q21;q34;q11.2)	-	47y/F	1/1	Similar to CML clinical features	Primary	-	Al-Achkar et al. 2010)
CML		-^**^	t(9;22;21)(q34;q11;p12)	-	36y/M	1/1	-	Primary	-	Al-Achkar et al. 2012)
Cranio-cerebello-cardiac (3C) syndrome		-^**^	t(12;17;18)(q21.2;q22;q21.1)	De novo	7 m/M	1/1	Craniofacial abnormalities including cleft palate, low set ears, hypertelorism, down-slanting palpebral fissures, depressed nasal bridge, and micrognathia	Complex translocation	-	Al-Achkar et al. 2011a)
CML		Rec^*^	t(9;12;19;22)	-	25y/F	1/1	Similar to CML clinical features	Complex with trisomy 8 and a derivative chromosome 12	-	Al Achkar et al. 2010a)
CML		Rec^**^	t(9;12;16;22)(q34;q24.2 ~ 24.31;p11.2;q11)	-	43y/F	1/1	CML in chronic phase	Complex	-	Al-Achkar et al. 2011b)
CML		Rec^*^	t(5;9;22)(p15.1; q34; q11.2)	-	-	1/1	-	Complex	-	Al-Achkar et al. 2007a)
CML		-^*^	t(1;4;5;9;22)(q42;p14;q31;q34;q11.2)	*-*	45y/F	1/1	CML in chronic phase	Complex	-	Al Achkar et al. 2009a)
ALL		-^**^	t(1;4;10)(1pter- > 1q42::4q21- > 4q35::10p15.3-10pter)	-	14y/M	1/1	B-cell ALL	Complex		Al Achkar et al. 2010b)
CML with primary myelofibrosis	Tunisia	-^**^	t(9;22;21)(q34;q11;q22)	-	67/M	1/1	CML with poor tyrosine kinase inhibitors (TKI) response	Associated with JAK2V617F mutation	-	Yamada et al. 2014)
APL		Rec^**^	t(12;15;17)(q24;q24;q11)	-	58y/M	1/1	APL (FAB-M4)	Complex	-	Bennour et al. 2013)
CML with variant Ph-rearrangements		Rec^*^ Rec^*^ Rec^**^ Rec^**^ Rec^**^ Rec^**^ Rec^**^ Rec^**^ Rec^*^ Rec^*^ Rec^*^ Rec^**^ Rec^**^ Rec^**^ Rec^*^ Rec^**^ Rec^**^ Rec^**^ Rec^**^ Rec^**^ Rec^**^ Rec^**^ Rec^**^	t(1;1;2;9;12;13;22)(q24;q31;p21;q34;q11.2) t(1;1;9;22)(p34;q42;q34;q11.2) t(1;9;22)(p35;q34;q11.2) t(1;9;22)(p36;q34;q11.2) t(10;9;22)(q25;q34;q11.2) t(11;9;22)(q12;q34;q11.2) t(3;9;22)(p14;q34;q11.2) t(3;9;22)(q26;q34;q11.2) t(4;9;22)(q13;q34;q11.2) t(4;9;22)(q27;q34;q11.2) t(4;9;22)(q34;q34;q11.2) t(6;9;22)(q21;q34;q11.2) t(6;9;22)(q22;q34;q11.2) t(9;12;12;22)(q34;q21;p12;q11.2) t(9;12;22)(q34;p13;q11.2) t(9;13;22)(q34;q13;q11.2) t(9;13;22)(q34;q31;q11.2) t(9;17;22)(q34;q22;q11.2) t(9;17;22)(q34;q23;q11.2) t(9;19;22)(q34;q13;q11.2) t(9;21;22)(q34;q22;q11.2) t(9;7;22)(q34;p21;q11.2) t(X;9;22)(p22;q34;q11.2)	-	-	23/336	Similar to CML clinical features	Associated with deletions	-	Bennour et al. 2009a)

**Abbreviations**: *Rec,* reciprocal; *Rob*, Robertsonian; *y*, years; *m*, months; *ALL*, acute lymphoblastic leukemia; *AML*, acute lymphocytic leukemia; *RPL*, recurrent pregnancy loss; *CML*, chronic myeloid leukemia; *APL*, acute promyelocytic leukemia

^*^Unique translocation, ^**^shared translocation, ^†^balanced translocation, ^‡^unbalanced translocation

### Clinical findings in Arab patients with CTs

**Fig. 3:**
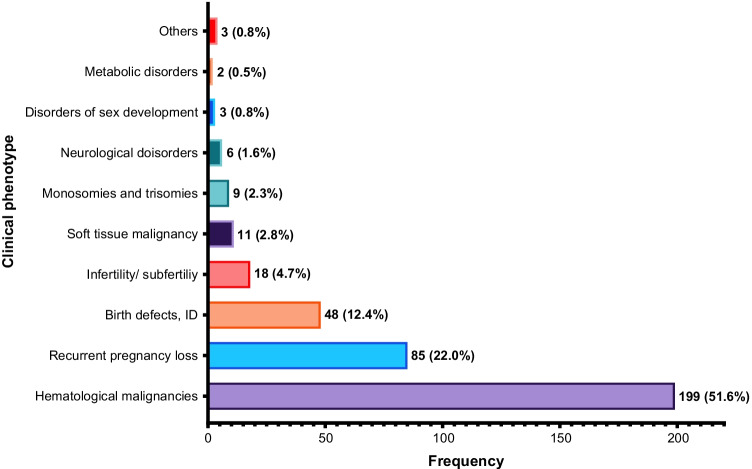
The clinical phenotypes associated with chromosomal translocations in the Arab countries. ID: intellectual disability

The clinical phenotypes associated with the reported CTs among Arabs are one or more of the following: hematological malignancies (51.6%), recurrent pregnancy loss (RPL) (22.0%), birth defects and intellectual disabilities (12.4%), infertility and subfertility disorders (4.7%), soft tissue malignancy (2.8%), monosomies and trisomies (2.3%), neurological disorders (1.6%), disorders of sex development (0.8%), metabolic disorders (0.5%), and other disorders (0.8%) as shown in Fig. 3. Hematological malignancies such as acute lymphoblastic leukemia (ALL), chronic myelogenous leukemia (CML), de novo acute myeloid leukemia (AML), multiple myeloma, and follicular lymphoma (FL) were the most reported malignancies among Arabs in Algeria, Egypt, Jordan, Kuwait, Lebanon, Morocco, Oman, Qatar, Saudi Arabia, Sudan, Syria, and Tunisia. Further, CTs related to recurrent pregnancy loss, birth defects, intellectual disabilities, monosomy, and trisomy syndromes were the most frequent disorders reported among patients from Egypt, Morocco, Oman, Saudi Arabia, and Tunisia (Table S1). Around 46% of the reciprocal translocations were reported in patients with malignancies, and 48% were associated with RPL, birth defects, and intellectual disability. In comparison, 80% of the Robertsonian translocations were reported in patients with RPL and intellectual and developmental disabilities (Table S1).

The clinical molecular diagnostics methods that were used to diagnose the CTs among Arab patients included FISH, karyotyping, multiplex PCR, RT-PCR, nested PCR, microarrays, immunophenotyping, western blot, northern blot, southern blot, immunohistochemistry, and comparative genomic hybridization (CGH) (Table S1).

### The frequency of unique CTs and their associated phenotypes among Arabs

Among the 378 identified CTs, 57 (15%) were unique to the Arab populations. The uniqueness of these CTs to Arabs was confirmed by searching these variants in different databases, including Mitelman Database, CytoD 1.0 Database, the Atlas of Genetics and Cytogenetics in Oncology and Hematology, PubMed, and Google Scholar. The highest number of identified unique CTs was found in Egypt (*n* = 12), followed by Tunisia (*n* = 10); Syria (*n* = 8); Morocco (*n* = 8); Saudi Arabia (*n* = 7); Qatar, Oman, and Kuwait (*n* = 3); Jordan (*n* = 2); and Lebanon (*n* = 1) (Table 1, Fig. 2). All identified distinctive CTs were reported only once among Arabs. Further, 12 were complex translocations involving more than two chromosomes.

As for the associated phenotypes, 23 (40.0%) of the identified unique CTs were found in patients diagnosed with hematological and soft tissue malignancies, mainly CML, AML, and FL, while 21 (35.6%) were found in patients with RPL, 8 (13.6%) in those with birth defects and intellectual disabilities, and four (6.8%) in those with fertility disorders (Table 1). Further, most of these unique CTs were reciprocal (67.8%) and associated with various conditions, whereas only three were Robertsonian (5.1%) and associated with RPL and fertility disorders.

### The frequency of complex CTs and their associated phenotypes among Arabs

**Fig. 4:**
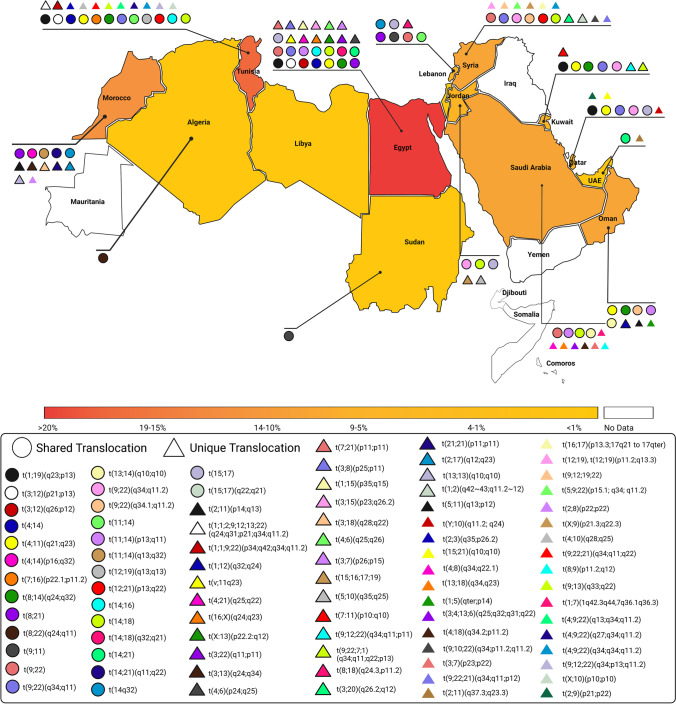
The distribution of chromosomal translocations in the Arab world. This figure was created with BioRender.com

Complex CTs involving more than two chromosomes were reported in 9 out of 15 Arab countries. As shown in Table 2, a total of 44 complex CTs were reported, of which 12 (27.3%) were unique to Arabs, and 32 (72.7%) were shared with other ethnicities. Tunisia had the highest number of reported complex CTs (*n* = 25), followed by Syria (*n* = 10), Kuwait, and Morocco (*n* = 2), while only one complex CT was reported in each of Algeria, Jordan, Lebanon, Oman, and Saudi Arabia. Among these complex CTs, 41 (93.2%) were associated with hematological malignancies, including CML (*n* = 35), AML (*n* = 3), and ALL and APL (*n* = 1). Further, 33 CTs involved three chromosomes (three-way CT), nine involved four chromosomes (four-way CT), and two involved five chromosomes (five-way CT). The t(4;9;22) and t(9;21;22) were the most frequently reported complex CTs in patients diagnosed with CML (*n* = 3 each) in Syria and Tunisia (Table 2, Fig. 4). Of note, 12 complex CTs were unique to the Arabs and not reported elsewhere.

### Distinctive phenotypes of shared CTs among Arabs

Out of 321 captured shared CTs, seven were reported with distinctive clinical phenotypes in the Arab patients, while they were associated with other clinical phenotypes in other ethnic groups. An example of such CTs is t(14;18), reported in a patient with chronic hepatitis C virus (HCV) infection in Egypt (Roulland et al. 2014). However, in the literature, this CT was reported in association with FL in Europe and East Asia (Zhu et al. 2020; Leich et al. 2009). Another example is t(12;19)(q13;q13), which is commonly reported in AML cases, but in Tunisia, this CT was reported in a patient with premature ovarian failure (Ayed et al. 2014; Huret et al. 2003). Further, t(7;16)(p22.1;p11.2) was reported with a distinctive phenotype in Tunisia in a patient with autistic disorder. In the literature and CTs databases, translocations between chromosome 7 and 16 at various breaking points were reported in cancer cases such as fibromyxoid sarcoma and endometrial stromal sarcoma, but no report of autistic disorders was found (t(7;16) n.d). Additionally, the t(1;16)(q23;q13), reported in Egypt in a case of cerebro-oculo-facio-skeletal (COFS) syndrome (Temtamy et al. 1996), have been associated with different phenotypes in other ethnic groups, such as malignant peripheral nerve sheath tumors (MPNST) in Japan (Velagaleti et al. 2004). Also, the t(3;4)(q28;p16), reported in Tunisia in a case of RPL (Hajlaoui et al. 2018a), was found to be linked with oropharynx squamous cell carcinoma in other ethnic populations (t(3;4)(q28;p16) n.d).

## Discussion

To our knowledge, this is the first review in the Arab world to comprehensively and systematically analyze peer-reviewed published articles related to patients with CTs from Arab countries. In this review, we investigated the spectrum and frequency of CTs in the Arab world. We used broad selection criteria to capture all data related to CTs in the Arab world. Our search strategy identified 168 studies, with a total of 378 CTs reported in 15 out of the 22 Arab countries (Table S1). The involvement of chromosomes in translocations showed a random distribution, where all the autosomal and sex chromosomes were involved in translocations at least on one occasion. The highest frequency of CTs was reported in Egypt, Tunisia, Morocco, Syria, and Saudi Arabia (Fig. 4). The captured CTs were detected using different molecular diagnostic methods but mainly using banded karyotyping, FISH, or RT-PCR. More recent studies used CGH to uncover cryptic rearrangements in the patients (Gregori et al. 2007; Vissers et al. 2007).

The most common CTs reported among Arabs were t(9;22), t(13;14), and t(14;18) at a frequency of *n* = 29, *n* = 15, and *n* = 13, respectively. The reciprocal CT t(9;22), was most reported in studies from Egypt, Syria, and Tunisia. This CT, which generates the Philadelphia chromosome (Ph), is usually detected in more than 90% of patients with CML and occurs in 3–5% of children with ALL, 25% of adult ALL, and in around 2% of children with AML (Aplenc et al. 2011; Aricò et al. 2000; Kang et al. 2016). A study conducted in Brazil reported a prevalence of 90.3% of classic Ph CT among CML patients (Chauffaille et al. 2015). Among Arabs, this translocation was primarily associated with hematological malignancies, including CML (44.8%), ALL (41.4%), and AML (10.3%). Notably, a higher prevalence of ALL due to Ph chromosome was reported among Arabs, particularly in male children aged 4–12 years. Although not reported, this could be due to the high prevalence of consanguinity and endogamy among Arabs and the major gaps between the social classes in the Arab countries compared to other populations (Tadmouri et al. 2009). A previous study conducted in the UAE suggested that socioeconomic factors could contribute to the relatively higher frequency of ALL among children of subcontinental origin when compared to other ethnic groups. In addition, the study indicated that parental consanguinity is significantly associated with the diagnosis of lymphomas among children (Révész et al. 1997; Révész et al. 1996).

Moreover, the Robertsonian CT, t(13;14), was the second most common CT reported among Arabs, showing the highest frequency in patients from Egypt (*n* = 5), Morocco (*n* = 4), and Tunisia (*n* = 3) (Fig. 4) with clinical phenotypes of RPL (*n* = 8) and fertility disorders (*n* = 4). This translocation is one of the most common Robertsonian CTs reported worldwide in which carriers usually show normal phenotypes, but male carriers can have infertility problems associated with oligospermia (Choi et al. 2013; Mahjoub et al. 2011). Female carriers of the karyotype 45,XX,t(13;14)(q10;q10) were reported to be at risk for developing reproductive problems, including miscarriage and infertility (Choi et al. 2013). A study conducted in Belgium reported a 66.7% prevalence of t(13;14) among Robertsonian CT carriers (Keymolen et al. 2011). In Poland, a cohort of 101 pedigrees of t(13;14) carriers was screened for clinical outcomes and showed a high frequency of recurrent miscarriage (34.7%) (Engels et al. 2008). However, no evidence of increased infertility rates among male and female carriers was found. Further, reports of this CT in children are scarce where only a single study conducted in Russia reported the occurrence of this CT in a child with developmental delay due to maternal inheritance (Dolskiy et al. 2018). Nevertheless, in this review, three children with Turner syndrome, Down syndrome, and intellectual disabilities were reported to have this CT in Egypt and Morocco (Mokhtar et al. 2003; Belkady et al. 2018; Latrech et al. 2018).

The t(14;18)(q32;21) was most frequently reported in patients with FL and diffuse large B-cell lymphoma. This reciprocal CT is considered a hallmark for FL and a recurrent abnormality in other types of non-Hodgkin lymphoma (NHL) (Rabkin et al. 2008). Further, the t(14;18) is frequently detected in the peripheral blood and tissue samples of healthy individuals, but the clinical significance is still unclear (Schüler et al. 2003). Additionally, the t(14;18)(q32;q21) is rarely associated with CLL and reported in less than 2% of CLL patients (Chen et al. 2016). Among Arabs, this CT was reported in a study conducted in Lebanon on a CLL Arab patient (Haddad et al. 2021). Tang et al. proposed that t(14;18)(q32;q21) could be an early pathogenetic event in CLL cases and may represent a secondary aberration that is not necessarily responsible for the disease onset since several CLL patients acquire novel abnormalities during the course of disease (Chen et al. 2016; Tang et al. 2013; Put et al. 2009; Shanafelt et al. 2006). Interestingly, two forms of this CT, t(14;18)(q13:p22) and t(14;18)(q21:p11), were reported in two females with RPL in Tunisia (Ayed et al. 2017b). A similar clinical phenotype was reported in only one study conducted in Japan on couples with two or more consecutive miscarriages, and hence, the exact involvement of t(14;18) in these cases remains unclear (Otani et al. 2006).

Among the identified CTs, 57 were distinctive to the Arab populations (Table 1) and were not previously reported in any study or database (Al-Achkar et al. 2013b, 2013c; Asif et al. 2016). All these CTs were reported once among Arabs, and hence, no frequent CTs were found in the Arab world. Interestingly, the t(21;21)(p11;p11) was the only CT reported with two different clinical phenotypes based on the type of translocation: intellectual disability when reciprocal and spontaneous RPL when Robertsonian (Belkady et al. 2018; Elkarhat et al. 2019). Both CTs were reported in 21-year retrospective studies conducted in Morocco on patients with intellectual disabilities and couples with recurrent spontaneous miscarriage, respectively.

Most of the unique CTs were identified in Egypt, which were mostly cases of RPL. Consanguinity was reported in only five cases (Elhady et al. 2020; AbouEl-Ella et al. 2018). However, it is most likely that consanguinity is underreported in these cases and could possibly be a significant contributor in RPL. Indeed, several studies conducted in Arab countries and non-Arab countries reported higher chances of miscarriage among consanguineous couples (Bellad et al. 2012; Saad and Jauniaux 2002; Gowri et al. 2011). The estimated prevalence of RPL is around 1–5% in married couples worldwide, where several etiological factors are involved, including parental chromosomal abnormalities (2–5%), anatomical alterations (10–15%), infections (0.5–5%), endocrinological disorders (17–20%), and immunological factors (20%) (Issa et al. 2021; Arias-Sosa et al. 2018). Nevertheless, in many cases, routine gynecological and laboratory investigations fail to identify the underlying cause of RPL. Hence, among the possible causes, CTs could be one of the etiological factors underlying RPL. Unfortunately, due to the growing cultural and religious sensitivity and controversy over reproductive health issues, this area remains relatively unexplored in Egypt. In addition, of the 57 unique CTs, 23 were detected in patients who presented with hematological malignancies and solid tumors, mainly in Syria and Tunisia (Table 1, Fig. 4). Previous studies reported that Syria had the highest incidence of leukemia at the national level in 2007 (Dong et al. 2020). This could be attributed to the unique CTs that have not been thoroughly investigated yet.

Complex CTs, which involve more than two breakpoints on two or more chromosomes, are not very common. However, among Arabs, complex CTs were reported in 11.6% of all identified CTs, mainly in Tunisia (56%) and Syria (23%), where chromosomes 9 and 22 were involved in 20 complex CTs. This CT was also reported in CML patients with complex variant translocations involving other chromosomes in addition to chromosomes 9 and 22 (Asif, et al. 2016; Manabe et al. 2011). However, such cases are not frequent and can be found in about 5–8% of CML cases (Manabe et al. 2011). Some studies have suggested that patients with variant Ph translocations may have an adverse prognosis (Gorusu et al. 2007; Potter et al. 1981; Loncarevic et al. 2002; Reid et al. 2003; Bernstein et al. 1984), while others suggested that these CTs have no prognostic effect (Bernstein et al. 1984; Marzocchi et al. 2011). Therefore, their impact on the prognosis and treatment response in CML patients is not conclusive. Notably, 12 complex CTs were not reported in the literature or any searched databases. Among these unique CTs, our search identified three-way, four-way, and five-way CTs, most of which involving the Ph chromosome and associated with CML and AML (Al-Achkar, et al. 2013c, 2007b; Kamal et al. 1996b; Adriana and Al Bahar 2012b; Achkar et al. 2010; Bennour et al. 2009b), except for one complex CT that was reported in a patient with a history of 12 miscarriages in Saudi Arabia (Turki et al. 2016b). Four-way CTs are rare, with less than 60 cases reported in the literature (Asif, et al. 2016). Similarly, five-way CTs are very rare in CML patients, with only a few cases reported (Yokota et al. 2012). Our search identified nine different four-way CTs, of which three were unique to Arabs and two five-way CTs, both of which were reported in CML cases in Syria and Tunisia and found to be unique to the Arab populations (Bennour et al. 2009b; Al Achkar et al. 2009b).

The findings of distinctive CTs and complex CTs could be due to the unique genomic architecture of Arabs (Zayed 2016b, 2016c), which is not well represented in the genomic databases. This emphasizes on the importance of such studies on the healthcare of Arab patients with CTs.

Finally, we investigated the clinical phenotypes of the shared CTs between Arabs and other ethnic groups; we further classified them as common or unique. We found that seven Arab patients seem to have manifested distinctive clinical phenotypes, mainly in Egypt and Tunisia. Nevertheless, no clear correlation between these CTs and the associated phenotypes was identified, which mandates further investigation to highlight the significance of these findings.

Some limitations were encountered in our study: first, the lack of detailed clinical data about the patients as most of the captured studies did not report some key data about the translocations, making it difficult to compare the different studies from different countries and identify other confounding factors that might be associated with the captured cases. Second, the variations in studies included in this review made it challenging to identify a general prevalence trend among Arab countries of CTs. Third, the lack of cytogenetics and molecular analyses in some studies; and fourth, variations in the detection methods used to capture the CTs, which could have affected the accuracy of the results in terms of identifying the exact breakpoints in the CTs. For instance, PCR-based detection was reported to be less sensitive than FISH analysis due to its inability to detect all breakpoint variants in CTs (Gomez et al. 2005). Therefore, a standard method of detection could help in improving the detection and diagnosis of CTs.

## Conclusion

This study addresses something that is not adequately reported, which is the ethnic CTs and their high relevance to cancer. This is the first systematic review to study the frequency and spectrum of CTs in the Arab region. In this study, 168 studies reported a total of 378 CTs in 15 Arab countries. We found distinctive CTs susceptibility profile to cancer and unique complex CTs that were found only among Arab populations (not existing in different ethnic groups); these are important for disease prognosis and diagnosis. This comprehensive study is important to highlight the health disparities that may exist within the Arab populations. Further, this work marks an important starting point for future studies focused on the etiology of CTs and highlights several hurdles within the Arab populations that will have to be overcome by further studies. This includes more openness and less stigma around issues such as reproductive health, consanguinity, and endogamy.

## Supplementary Information

Below is the link to the electronic supplementary material.

Supplementary file1 (DOCX 207 kb)
