# Supplementary material for: The spectrum of chromosomal translocations in the Arab world: ethnic-specific chromosomal translocations and their relevance to diseases
Source: Chromosoma. 2022 Jul 30;131(3):127–46. doi: 10.1007/s00412-022-00775-2 (PMC9470631; doi:10.1007/s00412-022-00775-2)
Supplement: Supplementary file 1 — Supplementary file1 (DOCX 207 kb) [file 412_2022_775_MOESM1_ESM.docx]

**The Spectrum of Chromosomal Translocations in the Arab World**

Hadeel T. Al-Jighefee^1,2^, Fatma H. Ali^2^, Hatem Zayed^1*^

*^1^Department of Biomedical Science, College of Health Sciences, Member of QU Health, Qatar University, P.O. Box 2713, Doha, Qatar*

*^2^Biomedical Research Center, Qatar University, P.O. Box 2713, Doha, Qatar*

**Correspondence: hatem.zayed@qu.edu.qa; Tel.: +974 4403 4809*

**Table S1**. The spectrum of chromosomal translocations (CT) among Arab countries.

| Disease | Arabic Country | Translocation’s type | Karyotype | Familial/ *De novo* | Age/ sex | No. of patients/ No. screened | Detection method | Clinical phenotype | Primary mutation/ associated with other abnormalities | Consanguinity | Reference |
| --- | --- | --- | --- | --- | --- | --- | --- | --- | --- | --- | --- |
| B-cell ALL | Algeria | Rec^**^ | t(14;14)(q11;q32) | *De novo* | 5y/F | 1/- | Kar and FISH | Pallor, cervical lymphadenopathy, poor prognosis, and frequent relapse | Associated with del(9)(p21) | - | [1] |
| Burkitt’s lymphoma |  | Rec^**^  Rec^**^  Rec^**^ | t(2;8)(p12;q24)  t(8;14)(q23 or q24;q32)  t(8;22)(q24;q11) | *De novo* | 9y/M  4y/M  3-54y/ 8M, 2F  4y/F | 2/2  10/10  1/1 | Kar | Enlarged lymph nodes, elevated WBCs count with absolute lymphocytosis, abdominal mass, splenomegaly, retroperitoneal adenopathy, hypercellular bone marrow | Primary | - | [2] |
| Burkitt’s lymphoma |  | Rec^**^ | t(2;8;9) | *De novo* | 9y/M | 1/22 | Cytogenetics | Jaw and abdominal tumors, facial asymmetry, enlarged lymph nodes and abdominal masses | A three-way recombination with translocation and insertion | - | [3] |
| Down syndrome |  | - | t(21;21)(q10;10) | - | -/M | 1/22 | Kar | Mental retardation and characteristic facial features | Primary | - | [4] |
| RPL with birth deformities | Egypt | Rec^†**^  Rec^†*^  Rec^†*^  Rec^†**^  Rec^†*^  Rec^†**^  Rob^**^ | t(11;22)(q23;q13)  t(3;8)(p25;p11)  t(4;6)(p24;q25)  t(6;12)(q21;q23)  t(7;21)(p11;p11)  t(9;17)(p24;q25.1)  t(9;21)(p21;q22)  t(13;14)(q10;q10) | **-**  *De novo*  **-**  **-**  **-**  **-**  **-**  *De novo* | 27y/M  30y/M  22y/F  31y/F  22y/F  24y/F  31y/M  25y/F | 1/-  1/-  1/-  1/-  1/-  1/-  1/-  1/- | Cytogenetics | Repeated abortions, stillbirth, fetal malformation, and birth of mentally handicapped children. | - | - | [5] |
| DiGeorge syndrome |  | Rec^†**^ | t(14;22)(q13;q11.2) | Fam | 0.7y/F  0.9y/F | 2/- | Cytogenetics | Developmental delay, mild dysmorphic features, recurrent infections, and congenital heart disease. | - | - | [6] |
| RPL |  | Rec^*^  Rec^*^  Rec^*^  Rec^*^  Rec^**^  Rec^**^  Rec^**^  Rec^**^  Rec^**^  Rec^**^  Rec^**^ | t(1;15)(p35;q15)  t(3;15)(p23;q26.2)  t(3;7)(p26;p15)  t(4;6)(q25;q26)  t(1;6)(q32.3;q26)  t(1;6)(q41;p24)  t(11;22)(q23;q11.2)  t(5;18)(q13.1;q12.2)  t(6;7)(q23;q13)  t(7;11)(q22;q23)  t(9;11)(q34;q23) | Fam | 23-50y/ 7F, 5M | 12/224 | Kar | Recurrent abortions, and the birth of dysmorphic/mentally handicapped infants | Primary | Consanguineous couple involving translocation in chromosomes 11 and 12 | [7] |
|  |  | Rob^**^  Rob^**^  Rob^**^ | t(13;14)(q10;q10)  t(14;15)(q10;q10)  t(21;22)(q10;q10) |  | 30-39y/ 2F, 2M | 4/224 |  |  |  |  |  |
| Primary tracheal synovial sarcoma |  | Rec^**^ | t(X;18)(p11;q11) | - | 26y/F | 1/1 | Cytogenetics and dc-FISH | Stridor, tracheal mass just proximal to the carina, neuroendocrine carcinoma | Primary | - | [8] |
| Monosomy 1p36 syndrome |  | -^‡**^ | t(1;21)(p36.32;q21.1) | *De novo* | 1.5y/F | 1/1 | Cytogenetics, microarrays, and FISH | Intellectual disability, delayed milestones, hypotonia, seizures, and dysmorphic features | Primary | No | [9] |
| Down syndrome |  | Rec^**^ | t(13q;21q) | *De novo* | -/2M,  2F | 4/712 | Cytogenetics | Similar to Down syndrome clinical phenotype, but could reduce the chances of conception and lead to miscarriages | Primary | Consanguinity was reported in 17.3% of the cases | [10] |
|  |  | Rob^**^  Rob^**^  Rob^**^ | t(14q;21q)  t(15q;21q)  t(21q;21q) |  | -/10M, 7F | 4/712  9/712  5/712 |  |  |  |  |  |
| Ovotesticular difference of sex development |  | -^**^ | t(X;Y)(p22.33;p11.32) | *De novo* | 11y/M | 1/530 | Kar and FISH | Ambiguous genitalia, gynecomastia, and short stature | Primary with unique karyotype comprising 3 different cell lines | No | [11, 12] |
| Missed abortion (silent miscarriage) |  | -^**^ | t(7:1)(q32:q23) | Mat | - | 2/60 of abortuses | FISH | Intrauterine fetal death during first trimester | Primary | - | [13] |
| Down syndrome |  | Rob^**^  Rob^**^  Rob^**^  Rob^**^ | t(13;14)  t(14;21)  t(21;21)  t(7;14) | Mat  *De novo,* Mat  Mat  Mat | - | 1/673  4/637  6/637  1/673 | Kar | Similar to Down syndrome clinical phenotype, but could reduce the chances of conception and lead to miscarriages | Primary | Consanguinity was reported in 17.3% of the cases. | [14] |
| 45,X testicular disorder of sex development |  | Rec^**^ | t(Y;14), t(Y;22) | Fam | 32y/M | 1/1 | FIS and multiplex PCR | Primary infertility, azoospermia, and Sertoli cell-only syndrome | Primary | - | [15] |
| ALL |  | Rec^**^  Rec^**^  Rec^**^  Rec^**^  Rec^**^, Rec^**^ | t(4;11)(q21;q23)  t(1;19)(q23;p13)  t(9;22)(q34;q11), t(3;12)(p21;p13)  t(8;14)(q24;q32)  t(11;14)(p13;q11), t(4;21)(q31;q22) | *-* | 5.5y/F  6m/M  3y/F  9y/M  10y/M  7y/F  12y/M | 3/41  1/41  1/41  1/41  1/41 | RT-PCR, FISH and immunophenotyping | t(4;11): high risk AML  t(1;19)(q23;p13): B-cell ALL  t(9;22): B-cell ALL  t(8;14): B cell ALL  t(11;14): T-cell ALL  t(4;21): T-cell ALL | Primary | - | [16] |
| Cerebro-oculo-facio-skeletal (COFS) syndrome |  | Rec^†**^ | t(1;16)(q23;q13) | Mat | 3y/F  31y/F | 2/3 | FISH | Intellectual disability, severe hypotonia, impaired reflexes, vision impairment, and involuntary eye movements. | Primary | - | [17] |
| Multiple myeloma |  | Rob^**^ | t(4;14) | *De novo* | Median: 46y (33-60)/ 12M, 5F | 17/80 | FISH | Multiple myeloma with poor prognosis | Associated with 17p deletion | - | [18] |
| AML |  | Rec^**^ | t(9;22)(q34;q11) | *De novo* | Median: 38y (25–55)/ 6M, 3F | 9/451 | Kar, FISH, and immunophenotyping | Clinical presentations are similar to that of AML, but Ph positivity in AML is more common in adult than children | Primary translocation | - | [19] |
| Biphenotypic acute leukemia |  | Rec^**^ | t(9;22)(q34;q11) | - | Median: 52y (38–61)/ 4M, 1F | 5/13 | Kar | Leukemia with poor prognosis | Primary | - |  |
| Therapy-related acute myeloid leukemias |  | Rec^*^ | t(v;11q23) | *De novo* | Media: 37y/ 46M, 28F | 6/120 | Immuno-phenotyping and cytogenetics | Poor topoisomerase II inhibitor treatment outcome | Primary | - | [20] |
| Lissencephaly with cerebellar hypoplasia |  | Rec^†**^ | t(7;12)(q22;p13) | Fam | -/ 1M, 1F | 2/2 | FISH and WB | Cortical lissencephaly with cerebellar hypoplasia, severe epilepsy, and mental retardation in homozygous children | Primary | - | [21] |
| ALL |  | Rec^**^ | t(4;21)(q28;q22) | *De novo* | 12y/M | 1/1 | FISH, RT-PCR, and NB | T-cell ALL | Associated with del(7)(q22) | - | [22] |
| Diffuse large B-cell Lymphoma |  | Rec^**^ | t(14;18)(q32;q21) | *De novo* | Mean: 50y (19-74)/ 5M, 3F | 8/40 | PCR and IHC | Non-Hodgkin lymphoma associated with failure to achieve a complete response to therapy and reduced survival | Associated with bcl-2 protein overexpression | - | [23] |
| Diffuse large B-cell Lymphoma |  | Rec^**^ | t(14;18)(q32;q21) | *De novo* | Median: 59y (39–71)/- | 11/26 | FISH and multiplex PCR | Non-Hodgkin lymphoma associated with failure to achieve a complete response to therapy and reduced survival | Associated with bcl-2 protein overexpression | - | [24] |
| Follicular lymphoma |  | Rec^**^ | t(14;18)(q32;q21) | *De novo* | Range: (36-78y)/ 11M, 7F | 14/18 | FISH and multiplex PCR | B-cell non-Hodgkin lymphoma | Associated with bcl-2 overexpression | - | [25] |
| AML |  | Rec^**^ | t(8;21) | *De novo* | - | 1/11  4/32 | Cytogenetics and dc-FISH | AML (M2) subtype  AML (M4) subtype | Associated with inv(16)/  t(16;16)(p13;q22) | - | [26] |
| Down syndrome |  | Rec^*^ | t(4;21)(q25;q22) | Mat | 8y/F | 1/1 | FISH | Severe growth retardation, microcephaly, hearing impairment, and specific facies | Associated with partial trisomy 4q25-qter and 21(pter-q22) | - | [27] |
| Chronic hepatitis C virus (HCV) infection |  | Rec^**^ | t(14;18) | *De novo* | - | 24/87 | PCR | B-cell lymphoproliferative disorders in chronic HCV infection | Associated with IgH rearrangement | - | [28] |
| ALL |  | -^**^  -^**^ | t(2;21)  t(9;22) | - | Range: (4-15y)/- | 9/40  2/40 | FISH | - | Associated with other abnormalities | - | [29] |
| RPL |  | Rob^†**^ | t(13;14)(q10;q10) | - | 26/M | 1/2 | G banding | Intrauterine fetal deaths (IUFDs) that involved multiple congenital anomalies | Double Robertsonian translocation | Yes | [30] |
| ALL |  | -^**^  -^**^  -^**^  -^**^  -^**^ | t(1;19)(q23;p13)  t(4;11)(q21;q23)  t(8;14)(q24;q32)  t(9;22)(q34;q11)  t(11;14)(p13;q11) | - | 3y/F  6m/M  10y/M  9y/M  7y/F | 1/41  2/41  1/41  1/41  1/41 | Cytogenetics, FISH, and RT-PCR | Pre-B ALL  Early pre-B ALL  B-cell ALL  Early pre-B ALL  T-cell ALL | Primary | - | [31] |
| ALL |  | -^**^  -^**^  -^**^  -^*^  -^**^  Rec^**^ | t(1;19)(q?23;p?13)  t(12;13)(q?12;q?13)  t(3;3)(p24;q13)  t(10;11)(p12−14;q21−q23)  t(8;14)(q24;q32)  t(9;22)(q34;q11) | - | 27/F  2y/M  34y/F  48y/F  65y/F  33y/M  14y/M  23y/M  49y/M  35y/M  51y/M  48y/F  35y/F  16y/M  11y/F | 1/25  1/25  1/25  1/25  2/25  9/25 | FISH and CISS | - | Associated with other abnormalities | - | [32] |
| AML |  | -^**^  -^**^  -^**^  -^**^  -^**^  -^**^  -^**^  -^**^ | t(1;5)(p11;q35), t(3;7)(q25;q22)  t(17;?)(p11.2;?)  t(3;12)(q26;p12)  t(3;3)(q21;q26)  t(4;12)(q12;p12–13)  t(8;12)(p21;p11.2)  t(8;22)(p11;q11)  t(9;22)(q34;q11), t(12;14)(q?14;q32) | *De novo* | 47y/M  50y/M  52y/F  48y/M  42y/F  23y/M  56y/M  51y/M | 8/25 | CGH | AML with poor prognosis | Associated with other abnormalities | - | [33] |
| Congenital anomalies |  | -^**^ | t(12;13)(q15;q34) | - | -/M | - | Kar | Congenital anomalies | - | - | [34] |
| RPL |  | -^**^  -^**^  -^*^  -^*^ | t(13;14)(q11;q11)  t(14;X)(q11;q12)  t(16;X)(q24;q23)  t(3;22)(q11;p11) | - | 32yr/M  26yr/F  39yr/M 6.5yr/M  38yr/M | 1/73  1/73  2/73  1/73 | Cytogenetic | Recurrent miscarriage | - | - | [35] |
| Congenital anomalies |  | -^**^ | t(2:9)(q21:q31) | - | - | - | Kar | Physical disabilities,   stillbirths and   neonatal deaths | - | consanguinity was positive in 43% of couples. | [36] |
| ADHD |  | -^**^ | t(4;8)(q13;q24.3) | - | 6.5yr/M | -/22 | Cytogenetic | Inattention, hyperactivity, and impulsivity | Associated with del7(q11;21;p22) | - |  |
| Congenital anomalies |  | -^*^ | t(X:13)(p22.2:q12) | - | - | - | Kar | Physical disabilities, stillbirths and neonatal deaths | - | consanguinity was positive in 43% of couples. |  |
| C syndrome/ Opitz trigonocephaly syndrome |  | Rec^†**^ | t(3; 18)(q13.13; q12.1) | - | 2.5yr/F | 1/1 | Kar | Trigonocephaly, variable mental retardation, hypotonia, variable cardiac defects, redundant skin, and dysmorphic facial features, including upslanted palpebral fissures, epicanthal folds, depressed nasal bridge, and low-set, posteriorly rotated ears | - | Non consanguineous | [37] |
| AML |  | -^**^  Rec^**^ | t(15;17)  t(8;21) | 45% of patients *De novo* | - | - | Cytogenetic and PCR | - | - | - | [38, 39] |
| Multiple myeloma |  | -^**^  -^**^ | t(14;16)  t(4;14) | - | - | - | FISH | - | Associated with deletion 13 and deletion of p54. | - | [40] |
| NHL | Jordan | Rec^**^ | t(14;18)(q32;q21) | *De novo* | Mean: 31.8y (19–69)/M | 61/96 | Nested PCR | NHL-Follicular lymphoma | Primary | - | [41] |
| AML |  | Rec^*^ | t(15;16;17;19) | - | 58y/F | 1/1 | FISH | Acute promyelocytic leukemia (AML-M3) | Primary complex translocation | - | [42] |
| AML |  | Rec^**^ | t(15;17) | - | 44y/F | 1/1 | FISH, RT-PCR, and DNA sequence analysis | AML-FAB M3 | Primary | - | [43] |
| AML |  | -^**^  -^**^ | t(11;17)(q23;q25)  t(15;17)(q22;q13) | *De novo* | Median 35.5y (16–49)/ 21M, 14F | 2/35  6/35 | Cytogenetics and immunophenotyping | AML-M5  AML-M3 | Primary | - | [44] |
| AML |  | Rec^**^ | t(9;22)(q34;q11) | - | 29y/F | 1/1 | CSF cytology | Spontaneous subdural hematoma after dasatinib treatment | Primary | - | [45] |
| Follicular lymphoma |  | -^**^ | t(14;18) | - | Mean: 53.2y (23-80)/- | 55/58 | Nested PCR | NHL | Primary | - | [46] |
| Unbalanced chromosomal rearrangement |  | -^*^ | t(5;10)(q35;q25) | Pat | 6m/F | 5/- | FISH | Developmental delay, hypotonia, supernumerary nipples, and distinct craniofacial features | Associated with der(10) | No | [47] |
| Translocation renal cell carcinomas (TFE3 RCC) |  | -^**^  -^**^  -^**^ | t(X;1)(p11.2;p34)  t(X;1)(p11.2;q21)  t(X;17)(p11.2;q25) | *De novo* | Mean: 35y  (2-58)/ 8M, 4F | 12/14 | IHC | Aggressive subtype of kidney cancer | Primary | - | [48] |
| NHL |  | -^**^ | t(14;18)(q32;q21) | - | ≥18/M | -/192 | PCR | - | - | - | [49] |
| AML | Lebanon | Rec^†**^ | t(12;19)(q13;q13) | - | 66y/F | 1/1 | Cytogenetics and SNP array | AML-M5 subtype with poor prognosis | Primary | - | [50] |
| MCL |  | -^**^ | t(11;14) | - | 57y/F | 1/1 | Concluded from clinical presentation | Primary gastrointestinal MCL with multiple lymphomatous polyposis | Associated with overexpression of Cyclin D1 | - | [51] |
| ALL |  | Rec^**^  Rec^**^  -^**^  -^**^ | t(9;22)  t(4;11)  t(1;19)  t(12;21) | - | Median: 34y  (18-94)/- | 21/148  8/148  4/148  2/148 | Cytogenetics | B-cell ALL | Primary | - | [52] |
| AML |  | Rec^**^  Rec^**^  Rec^**^ | t(15;17)  t(8;21)  t(9;11) | *De novo* | Mean: 50y  (18-89)/ 325M, 255F | 44/580  24/580  4/580 |  | AML | Primary | - |  |
| CLL |  | -^**^ | t(14q32) | - | - | 7/28 | Cytogenetics and FISH | CLL with good prognosis | Primary | - | [53] |
| CML |  | Rec^**^ | t(9;22) | *De novo* | Median: 14y  (6‐17)/ 120M, 181 F | 6/301 | Cytogenetic and molecular analysis | Do not have a significant prognostic impact | Primary | - | [54] |
| Follicular lymphoma |  | Rec^**^ | t(14;18)q(32;21) | - | - | 19/42 | PCR | - | Primary | - | [55] |
| AML |  | -^**^ | t(8;12;21)(q22;p12 approximately p13;q22) | - | 32y/M | 1/1 | dc-FISH and RT-PCR | AML (FAB- M2) | Associated with chromosomal abnormalities (loss of Y ch. and trisomy 8q22) | - | [56] |
| Constitutional jumping translocations |  | -^*^ | t(8;18)(q24.3,p11.2) | - | 26y/M | 1/1 | FISH | Partial hypogonadism | Associated with ring chromosome 18 | - | [57] |
| AML |  | -^**^ | t(8;21) | - | ≤10y/- | - | Cytogenetics and molecular analysis | AML | - | - | [58] |
| CLL |  | -^**^ | t(14;18) | - | 53y/F | 1/1 | Kar and FISH | Common type of adult leukemia | Associated with an extra chromosome 12 (trisomy chromosome 12) | - | [59] |
| Spontaneous RPL | Kuwait | -^†*^ | t(7:11)(p10:q10) | *De novo* | 37y/F | 1/1 | Cytogenetics | High-order miscarriage | Associated with other etiological factors | No | [60] |
| CML |  | Rec^**^  Rec^**^ | t(16;16)(p13;q22)  t(9;22)(q34;q11.2) | - | 37y/M | 1/1 | Cytogenetics | Blastic CML, cervical lymphadenopathy, hepatomegaly, and splenomegaly | Associated with del(7q) | - | [61] |
| CML |  | Rec^*^ | t(9;22;12)(q34;q11;p11) | - | 26y/M | 1/1 | FISH | Similar to CML clinical features | Primary | - | [62] |
| CML |  | -^*^ | t(9;22;7;1)(q34;q11;q22;p13) | - | 64y/M | 1/1 | Cytogenetic and molecular analyses | Similar prognosis to those with classical Ph translocations | Associated with tyrosine kinase inhibitor therapy | - | [63] |
| ALL |  | -^**^  -^**^  -^**^  -^**^  -^**^  -^**^ | t(8;22)(q24;q11)  t(1;19)(q23;p13)  t(1;7)(q31;p22)  t(4;11)(q21;q23)  t(8;14)(q24;q32)  t(9;22)(q34;q11) | - | 12yr/M  6yr/F  14yr/M  9yr/M  11yr/M  4yr/M | 6/- | Cytogenetic and G-banding | ALL | Associated with t(4;11)(q21;q23) | - | [64] |
| Congenital cataract | Libya | Rec^**^ | t(3;5)(p22.3; p15.1) | Fam | - | 6/13 | Cytogenetics | Visual impairment or blindness in children | Primary | - | [65] |
| CML | Morocco | -^**^ | t(9;18;22)(q34;p11;q11) | - | 29y/M | 1/1 | dc-FISH | Similar to CML clinical features | Associated with der(18) | - | [66] |
| Intellectual disability |  | Rec^**^  Rec^*^  Rec^*^  Rec^**^  Rec^**^  Rec^**^ | t(13;14)(p11;p11)  t(2;17)(q12;q23)  t(21;21)(p11;p11)  t(1;6;7)(p21;q16;p21)  t(14;21)(p11;p11)  t(14;21)(q10;q10) | - | -/8M, 6F | 14/1200 | Kar | Non-syndromic intellectual disability | Primary | - | [67] |
|  |  | Rob^**^ | t(14;21)(q10;q10) | - | - | 1/12000  34/1200 |  |  |  |  |  |
| Spontaneous RPL |  | Rec^*^  Rec^*^  Rec^*^  Rec^*^  Rec^**^  Rec^**^  Rec^**^  Rec^**^  Rec^**^  Rec^**^  Rec^**^  Rec^**^  Rec^**^  Rec^**^  Rec^**^  Rec^**^  Rec^**^ | t(2;11)(p14;q13)  t(2;8)(p22;p22)  t(3;13)(q24;q34)  t(3;18)(q28;q22)  t(6;11)(p21;q21)  t(1;18)(q11;p11)  t(1;7)(p16;q11)  t(1;9)(q41;p23)  t(13;19)(q33;q11)  t(14;15)(q11;q23)  t(14;17)(q33;q24)  t(15;21)(q24;q22)  t(5;10)(p15;q26)  t(6;10)(q22;q23)  t(7;8)(p21;p12)  t(8?;8?)(p22?;q23?)  t(9;10)(q31;q25) | - | -/7M, 10F | 17/1254 | Cytogenetics | Recurrent spontaneous miscarriage | Primary | - | [68] |
|  |  | Rob^**^  Rob^**^  Rob^*^  Rob^**^  Rob^*^  Rob^**^  Rob^*^ | t(13;14)(p11;p11)  t(13;14)(q10;q10)  t(21;21)(p11;p11)  t(21;21)(q10;q10)  t(13;13)(q10;q10)  t(13;22)(p11;p11)  t(15;21)(q10;q10) | - | -/2M, 7F | 9/1254 |  |  |  |  |  |
| ALL |  | Rec^**^ | t(14;21)(q11;q22) | - | 13y/M | 1/70 | Cytogenetics and FISH | T-cell ALL | Primary | - | [69] |
| Multiple myeloma |  | -^**^ | t(14q32) | - | Median: 55y/ 6M, 5F | 11/93 | Cytogenetics and FISH | Multiple myeloma with neutral/ adverse prognosis | Primary in 6 patients, 5 associated with other abnormalities | - | [70] |
|  |  | -^**^ | t(4;14)(p16;q32) | - | Median: 62y/ 8M, 5F | 13/93 |  | Multiple myeloma with adverse prognosis |  | - |  |
| Turner syndrome |  | Rob^**^ | t(13;14) | *De novo* | 3m/F | 1/1 | Cytogenetics | Gonadal dysgenesis, Mullerian ducts and palpable gonad in inguinal position and low hairline | Primary | No | [71] |
| Pregnancy-CML |  | Rec^**^ | t(9,22)(q34;q11.2) | - | 37y/F | 1/1 | Cytogenetics | CML progression (relapse with blast crisis) after the completion of 36 weeks of gestation | Primary | - | [72] |
| Sensorineural deafness |  | -^‡**^ | t(X;Y)(p22;q12) | Mat | 6y/M | 3/4 | Kar, FISH, and aCGH | Peculiar gestalt, short stature, and hearing loss, | Associated with other abnormalities | Yes | [73] |
| Down syndrome |  | Rob^**^  Rob^**^  Rob^**^  Rob^**^ | t(13;21),+21  t(14;21),+21  t(21;21),+21  t(22;21),+21 | 6 Fam, 21 *De novo* | - | 27/852 | Kar | Down syndrome associated with advanced maternal age | Primary | - | [74] |
| ALL |  | Rec^**^ | t(14;21)(q11;q22) | - | 2m/- | 1/1 | Kar | - | Primary | - | [75] |
| Multiple myeloma |  | -^**^  -^**^ | t(11;14)(q13;q32)  t(4;14)(p16;q32) | - | 68y/F | 1/1 | Kar | - | Associated with del(5)(q13q33),  del(6)(q13q23),  del(8), del(17p)  and add (1q) | - | [76] |
| AML |  | -^**^ | t(8;21) | - | 25yr/- | -/85 | Kar | - | - | - | [77] |
| AML | Oman | -^**^ | t(8;13;21)(q22;q14;q22) | - | 33y/F | 1/1 | dc-FISH | AML-FAB M2 | Primary | - | [78] |
| ALL |  | -^**^  -^**^  -^**^  -^**^  -^**^  -^**^ | t(1;19)(q23;p13.3)  t(11;14)(p13;q11)  t(12;21)(p13.3;q22)  t(4;11)(q21;q23)  t(8;14)(q24;q32)  t(9;22)(q34.1;q11.2) | - | - | 3/93  1/93  5/93  2/93  3/93  4/93 | FISH | ALL B-cell (L1)  ALL T-cell  ALL L3 (BL)  ALL in children  ALL with goof prognosis  ALL with poor prognosis | Primary | - | [79] |
| RPL |  | Rec^*^  Rec^*^  Rec^**^  Rec^**^  Rec^**^  Rec^**^  Rec^**^  Rec^**^  Rec^**^  Rec^**^  Rec^**^  Rec^**^  Rec^**^  Rec^**^  Rec^**^  Rec^**^  Rec^**^  Rec^**^ | t(1;12)(q32;q24)  t(1;5)(qter;p14)  t(2;10)  t(1;12)(q42;q24)  t(1;6)(q25;q16)  t(10;11)(p13;q13)  t(10;18)(p12;p11.2)  t(10;18)(p13;q21.1)  t(10;18)(p13;q23)  t(16;18)(p12;q23)  t(4;18)(q22;q23)  t(5;10)(q35;q24)  t(7;14)(q22;p10)  t(7;18)(q22;p11)  t(7;20)(p15;q13)  t(8;18)(q23;q22)  t(8;21)(p11.2;p11)  t(X;6)(q22;q15) | - | 29y/M  29y/M  25y/F  35y/M  33y/F  28y/F  25y/F  30y/M  34y/M  35y/M  29y/F  35y/F  26y/F  36y/F  30y/F  26y/F  43y/M  20y/F | 18/760 | Kar | Miscarriage occurrence of at least two times | Primary | - | [80] |
|  |  | Rob^**^  Rob^**^  Rob^**^ | t(13;14)(q10;q10)  t(13;14)(q10;q10)  t(13;22)(q10;q10) | - | 23y/F  25y/F 24y/F | 3/760 |  |  |  |  |  |
| ALL |  | -^*^  -^**^ | t(5;11)(q13;p12)  t(X;6)(p22;q23) | - | 0.7y/M | 93/120 | Cytogenetic | Pre-B ALL | - | - | [81] |
| Partial trisomy 13q syndrome | Palestine | Rob^**^ | t(8;13)(p23;q14) | *De novo* | 7m/M | 1/1 | Kar | Dysmorphic features | Primary | - | [82] |
| APL | Qatar | -^**^ | t(15;17) | - | Median: 38y (16-56)/- | 5/10 | Cytogenetics | 1 classic rare, 2 M3 variant, 2 hyper basophilic APL | Primary | - | [83] |
| CML |  | Rec^**^ | t(9;22)(q34;q11.2) | - | 45y/M | 1/1 | FISH | CML with extramedullary B-lymphoid blast phase | Associated with other abnormalities | - | [84] |
| Acute nonlymphocytic leukemia |  | Rec^**^ | t(7;22)(p22;q13) | *De novo* | 20m/F | 1/1 | Kar | ANLL- FAB M4 | Associated with inv(16)(p13q22) | - | [85] |
| ALL |  | -^**^  -^**^  -^**^  -^**^  -^**^  -^**^  -^**^ | t(1;19)(q23;p13)  t(11;19)(q23;p13)  t(12;21)(p12;q22)  t(4;11)(q21;q23)  t(7;9)(q34;q34.32)  t(9;11)(p21-22;q23)  t(9;22)(q34;q11) | - | Mean: 6.1y (0.2-18)/- | 16/259  1/89  61/416  24/464  1/77  1/99  25/491 | Conventional cytogenetics, FISH, and PCR | - | Primary | - | [86] |
| CML |  | Rec^**^ | t(9;22)(q34; q11.2) | - | 57y/M | 1/1 | Kar and FISH | CML with good hematological and cytogenetic response to dasatinib | Associated with JAK2 V617F mutation | - | [87] |
| Azoospermia and Severe oligozoospermia |  | Rec^*^  Rec^*^  Rec^**^  Rec^**^  Rec^**^  Rec^**^ | t(2;9)(p21;p22)  t(Y;10)(q11.2; q24)  t(11;22)(p10;q10)  t(2;4)(P11.2;q31.3)  t(11;19)(p10;p10)  t(5;6)(p10;q10) | - | -/M | 49/511 | FISH | Azoospermia, severe oligozoospermia and infertility in men | - | - | [88] |
|  |  | Rob^**^  Rob^**^  Rob^**^  Rob^**^ | t(13;14)(q10;q10)  t(14;15)(q10;q10)  t(15;21)(q10;q10)  t(14;21)(q10;q10) | - | -/M |  |  |  |  |  |  |
| FOXA2 mutation |  | Rec^**^ | t(6;20)(p11; p11) | *De novo* | 6y/M | 1/1 | Kar | Associated with multiple phenotypes including developmental delay, panhypopituitarism, heterotaxy and neurodevelopmental abnormalities, hypopituitarism, hyperinsulinism, endodermal organ and craniofacial abnormalities. | Associated with del (20) (p11.21p11.22) | - | [89] |
| RPL | Saudi Arabia | Rob^**^  Rec^*^  Rec^*^  Rec^**^ | t(14:21)(q11.1;q11.1)  t(3;4;13;6)(q25;q32;q31;q22)  t(3;7)(p23;p22)  t(7;14)(q34;p10) | - | 30y/F  -/M  33y/F  34y/F | 1/171  3/171 | Cytogenetics | High average of pregnancy failures | Associated with Factor V Leiden and Prothrombin A20210G allelic polymorphisms. | 33 % of couples had family history of consanguineous marriages. | [90] |
| ALL |  | -^**^ | t(12,21) | - | Median: 5y (0.5-15)/- | 31/213 | Cytogenic and molecular analysis | B-cell ALL | Primary | - | [91] |
| Renal cell carcinoma |  | -^**^ | t(Xp11.2) | - | <60y/ 4M, 1F | 2/44 | FISH, and IHC | Large sized and higher grade | Primary | - | [92] |
| Unbalanced inherited translocation |  | Rec^*^  Rec^**^  Rec^*^ | t(1;7)(1q42.3q44,7q36.1q36.3)  t(1;7)(q44;q36)  t(1;7)(1q42.3q44,7q36.1q36.3) | Mat | - | 3/5 | aCGH | Neurological phenotype and brain malformation | Primary | Yes | [93] |
| Infertility |  | Rob^**^ | t(13;14) (q10;q10) | - | 34y/M | 2/2 | Cytogenetics | Cryptozoospermia | Primary | - | [94] |
| Type 1 diabetes |  | Rec^**^  Rec^*^ | t(3;12)(p14.2;q21.2)  t(4;18)(q34.2;p11.2) | -  Pat | 53y/M  21y/M | 4/7  3/7 | Kar | Microcephaly, ectodermal dysplasia, hepatosplenomegaly | Associated with -18, +der (18) | Yes | [95] |
| Desmoplastic small round cell tumor |  | Rec^**^ | t(11;22)(p13;q12) | - | 9y/M | 1/1 | RT-PCR | Abdominal mass of pancreatic origin and very poor prognosis | Associated with EWSR1-WT1 gene fusion | - | [96] |
| Desmoplastic small round cell tumor |  | Rec^**^ | t(11;22)(p13;q12) | - | 26y/F | 1/1 | FISH | Epigastric mass and very poor prognosis | Primary | - | [97] |
| Pure erythroid leukemia |  | -^*^ | t(8;9)(p11.2;q12) | - | 48y/M | 1/1 | Cytogenetics | Pancytopenia and  circulating erythroblast in peripheral blood | Associated with del(5q) and del(7q) | - | [98] |
| Mantle cell lymphoma |  | -^**^ | t(11;14)(q13;q32) | *De novo* | 64y/M | 1/1 | Kar and FISH | Advanced CD5- MCL  with blastoid variant  and massive bone marrow necrosis | Associated with other abnormalities | - | [99] |
| CML with vision loss |  | Rec^**^ | t(9;22) | *De novo* | 36y/M | 1/1 | FISH | Progressive loss of vision | Primary | - | [100] |
| Monosomy 21 anomaly |  | -^**^ | t(5p;21q) | *De novo* | 6m/M | 1/1 | FISH | Dysmorphic features | Primary | Yes | [101] |
| CHARGE syndrome |  | -^*^ | t(4;8)(q34;q22.1) | *De novo* | 2m/M | 1/1 | FISH and aCGH | choanal atresia, facial dysmorphism, cardiovascular malformations, and developmental delay | Primary | No | [102] |
| Clear cell sarcoma |  | -^**^ | t(12;22)(q13;q12) | - | 25y/F  19y/F | 2/2 | Cytogenetics | Malignant melanoma of soft tissue | Primary | - | [103] |
| Unbalanced translocation |  | Rec^**^ | t(2;8)(p23;p23.2) | - | 11m/F | 1/1 | Cytogenetics | Severe hypercalcemia | Primary | No | [104] |
| Syncytial meningioma |  | -^**^ | t(3;9)(q13.3;q22) | *De novo* | 35y/M | 1/1 | Cytogenetics | Brain tumor in | Associated with other abnormalities | - | [105] |
| Follicular cell lymphoma |  | -^**^ | t(14;18) | - | - | 10/10 | PCR and SB | - | Primary | - | [106] |
| Intellectual disability |  | -^‡*^ | t(13;18)(q34,q23) | *De novo* | 13y/M  11y/M | 2/2 | Kar and aCGH | Intellectual disability, obesity, dysmorphic  features, speech delay, and seizure | Associated with 13q34 microdeletion, 18q23 microduplication, and 6q25 deletion | - | [107] |
| Plasma cell leukemia |  | -^**^  -^**^ | t(8;14)(p21;q32)  t(12;22)(p13;q13) | - | 52y/F | 1/1 | Cytogenetic | Intellectual disability, obesity, dysmorphic features, speech delay, and seizure | Associated with add(19)(q13.4) | - | [108] |
| Ewing’s sarcoma |  | -^**^ | t(11;22)(q24;q12) | - | 15y/F | 1/1 | - | Rare malignant tumor of the primitive neuroectodermal  tumor family | - | - | [109] |
| Klinefelter Syndrome |  | -^**^ | t(4,11)(q35,q23) | - | 28y/M | 1/1 | Kar | Associated to multiple phenotypic features including cardiac abnormalities, infertility, mental retardation, diabetes, and increased cancer risk | - | - | [110] |
| Rett syndrome |  | -^**^ | t(1;7)(p13.3;q31.3) | - | -/F | 2/2 | - | Derangements in nervous system | - | - | [111] |
| Dermatofibrosarcoma protuberans |  | -^‡**^ | t(17;22)(q21;q13) | - | 30y/F | 1/1 | - | Rare fibroblastic mesenchymal skin tumor | - | - | [112] |
| ALL |  | Rec^**^ | t(9;22)(q34;q11) | - | - | - | Cytogenetic | - | - | - | [113] |
| Hybrid cell acute leukemia |  | - | t(11:17);(q23;p13) | - | 16d/M | 1/1 | Cytogenetics | leukemic cells co-expressing myeloid and lymphoid B cell antigens | - | - | [114] |
| AML | Sudan | Rec^**^  Rec^**^  Rec^**^ | t(16;21)(p11;q22)  t(8;21)(q22;q22)  t(9;11) | - | Mean: 36y (2–84)/1.15 M:1 F | 7/97  56/97  34/97 | RT-qPCR | AML with different FAB classifications | Primary | - | [115] |
| Pre-B cell ALL | Syria | -^**^ | t(1;19)(q21.1;p13.3) | *De novo* | 15y/M | 1/1 | Kar | B-call ALL with high incidence of central nervous system relapse | Associated with deletion 6q12 to 6q26 and der(13) | - | [116] |
| RPL |  | Rob^**^ | t(22;22)(q10;q10) | *De novo* | 29y/F | 1/1 | Kar | RPL | Primary | - | [117] |
| APL |  | Rec^*^  Rec^**^ | t(1;2)(q42~43;q11.2~12)  t(15;17)(q22;q21) | *De novo* | 46y/F | 1/1 | Kar, FISH | Multiple sclerosis, fatigue, loss of weight, fever, and an elevated WBC count | Two associated translocations | - | [118] |
| CML |  | Rec^**^ | t(9;11;20;22)(q34;p11.2;q11.21;q11) | - | 55y/F | 1/1 | aMCB | No symptoms were observed, but the patient was lost during follow-up | Primary | - | [119] |
| CML |  | Rec^*^ | t(9;10;22)(q34;p11.2;q11.2) | - | 42y/M | 1/1 | Kar and FISH | Imatinib mesylate-resistant CML | Primary | - | [120] |
| CML |  | -^**^ | t(1;2;9;22)(p32;q21;q34;q11.2) | - | 47y/F | 1/1 | Kar and FISH | Similar to CML clinical features | Primary | - | [121] |
| AML |  | -^**^ | t(3;12)(q26;p12) | *De novo* | 63y/F | 1/1 | Kar, FISH, and RT-PCR | AML-FAB M4 | Associated with trisomy 8 | - | [122] |
| CML |  | -^**^ | t(7;8)(p11.2:q11.2) | - | 22y/F | 1/1 | Kar, FISH, and RT-PCR | Imatinib mesylate-resistant CML | Primary | - | [123] |
| Down syndrome associated AML |  | -^**^  -^**^ | t(3;20)(q25;q13.1)  t(1;16)(q25.3;q24) | - | 1.4y/M | 1/1 | Cytogenetic and molecular analysis | AML-M2  AML-M1 | Associated with trisomy 22  Associated with trisomy 21 | - | [124] |
| CML |  | -^**^ | t(9;22;21)(q34;q11;p12) | - | 36y/M | 1/1 | FISH and aMCB | - | Primary | - | [125] |
| CML |  | Rec^**^  Rec^*^ | t(9;22)(q34;q11)  t(16;17)(p13.3;17q21 to 17qter) | - | 30y/M | 1/1 | G banding, molecular cytogenetics, dc- FISH and aMCB | CML with complex secondary chromosomal changes, treatable with Imatinib | Associated with partial trisomy of 17q21 to 17qter and trisomy 9 | - | [126] |
| Cranio-cerebello-cardiac (3C) syndrome |  | -^**^ | t(12;17;18)(q21.2;q22;q21.1) | *De novo* | 7m/M | 1/1 | G banding, M-FISH, and MCB | Craniofacial abnormalities including cleft palate, low -set ears, hypertelorism, down-slanting palpebral fissures, depressed nasal bridge, and micrognathia | Complex translocation | - | [127] |
| CML |  | Rec^*^  Rec^*^ | t(12;19), t(12;19)(p11.2;q13.3)  t(9;12;19;22) | - | 25y/F | 1/1 | G banding, dc-FISH, and aMCB | Similar to CML clinical features | Complex with trisomy 8 and a derivative chromosome 12 | - | [128] |
| CML |  | Rec^**^ | t(9;22)(q34.1;q11.2) | - | 62/M | 1/1 | G banding, FISH and MCB | CML in clast crisis after Imatinib treatment | Complex with inv dup(22)(q11.23), tetrasomy 8 and trisomy 19 | - | [129] |
| CML |  | Rec^**^ | t(9;12;16;22)(q34;q24.2~24.31;p11.2;q11) | - | 43/F | 1/1 | G banding, dc-FISH, and aMCB | CML in chronic phase | Complex | - | [130] |
| CML |  | Rec^**^ | t(9;22)(q34;q11.2) | - | 53/M | 1/1 | G banding, dc-FISH, and PCR | CML in acceleration phase | Primary with hyperdiploidy | - | [131] |
| CML |  | Rec^*^ | t(5;9;22)(p15.1; q34; q11.2) | - | - | 1/1 | G banding and dc-FISH | - | Complex | - | [132] |
| ALL |  | -^**^  -^**^  Rec^**^ | t(12;14)(q12;p12)  t(2;20)  t(9;22)(q34;q11) | *De novo* | 31y/F | 1/1 | G banding and dc-FISH | Adult B-cell precursor ALL with multiple secondary cytogenetic aberrations | Multiple translocations with monosomy 9 | - | [133] |
| Follicular lymphoma and B-cell lymphoblastic leukemia |  | -^**^  -^*^  -^**^  -^**^  -^*^ | t(2;9)(p21;q22.3)  t(3;20)(q26.2;q12)  t(6;14)(p22.3;q32)  t(X;20)(p21.3;q11.2)  t(X;9)(p21.3;q22.3) | *De novo* | 38/F | 1/1 | G banding and mc-FISH | Adult FL grade 2 transformed to B-ALL | Complex | - | [134] |
| CML |  | -^**^ | t(1;4;5;9;22)(q42;p14;q31;q34;q11.2) | *-* | 45y/F | 1/1 | G banding and dual-color FISH | CML in chronic phase | Complex | - | [135] |
| ALL |  | -^**^ | t(9;22)(q34;q11.2)  t(1;4;10)(1pter->1q42::4q21->4q35::10p15.3-10pter)  t(9;22)(q34;q11.2) | - | 14y/M | 1/1 | G banding, FISH, and aMCB | B-cell ALL | Complex |  | [136] |
| CML |  | -^**^  Rec^**^ | t(10;17)(q22.3;p13.1)  t(9;22)(q34;q11) | - | 39/F | 1/1 | G banding and tri-color FISH | - | Complex CT associated with inversion in chromosome 10 | - | [137] |
| RPL |  | Rec^**^  Rob^**^ | NA | - | Mean: 30.6y/- | 36/2148  8/2148 | Cytogenetics | Frequent miscarriage | Primary and associated with other etiological factors | - | [138] |
| Unbalanced spermatozoa | Tunisia | Rob^**^ | t(13;14) | - | Mean: 39y (36-41) | 5/5 | Tri-color FISH | Infertility | Primary | - | [139] |
| RPL |  | Rob^**^  Rec^**^  Rec^**^  Rec^*^ | t(13;14)(q10;q10)  t(14;18)(q13:p22)  t(14;18)(q21:p11)  t(4;10)(q28;q25) | - | 31y/F  -/F | 1/326  3/326 | Kar | Recurrent miscarriage | Primary | - | [140] |
| Miller-Dieker syndrome |  | Rec^**^ | t(3,17)(p26.2;p13.3) | Mat | 2y/F  4m/M | 2/2 | FISH and aMCB | Lissencephaly | Primary | - | [141] |
| Autism |  | Rec^**^ | t(7;16)(p22.1;p16.2) | *De novo* | -/M | 1/1 | Kar, FISH, and qPCR | Not associated with any physical abnormalities | Primary | No | [142] |
| CML |  | Rec^**^ | t(9;22)(q34;q11) | - | - | 301/336 | R banding and FISH | - | Primary | - | [143] |
| CML |  | Rec^**^ | t(9;22)(q34;q11) | - | 27y/M  46/M  14/F | 3/3 | Multiplex RT-PCR and FISH | Similar to CML clinical features | Primary | - | [144] |
| CML |  | Rec^**^ | t(9;22)(q34;q11) | - | - | 45/60 | Multiplex RT-PCR and conventional cytogenetics | Similar to CML clinical features | Primary | - | [145] |
| Multiple Myeloma |  | -^**^  -^**^  -^**^ | t(11;14)  t(14;16)  t(4;14) | - | Median: 56y/ 37M, 33F | 32/70  2/70  8/70 | R banding and FISH | - | Associated with other chromosomal abnormalities | - | [146] |
| CML with primary myelofibrosis |  | -^**^ | t(9;22;21)(q34;q11;q22) | - | 67/M | 1/1 | Kar, FISH, and PCR | CML with poor tyrosine kinase inhibitors (TKI) response | Associated with JAK2V617F mutation | - | [147] |
| Prader-Willi syndrome |  | -^**^ | t(X;3)(q27.3; p26.3) | Mat | 24m/M | 1/1 | FISH, PCR, and aCGH | Prader-Willi like features and hypotonia (severe psychomotor retardation, facial dysmorphic features with a broad face, a small mouth and a thin pointed nose, urogenital malformation, and proneness to infections) | Associated with deletion 3p26.3 | - | [148] |
| Accelerated CML |  | Rec^**^  Rec^**^ | t(3;12)(q21;p13)  t(9;22)(q34;q11) | - | 28/M | 1/1 | RHG banding and FISH | Accelerated chronic myeloid leukemia after imatinib and nilotinib therapy | Associated with 8p21.3 deletion | - | [149] |
| Partial  monosomy 10q and partial trisomy 11q |  | -^**^ | t(10;11)(q26;q13) | *De novo* | 6m/M | 1/1 | R banding | Craniofacial dysmorphy, congenital heart defects, urogenital and cerebral anomalies, and severe developmental delay | Associated with monosomy 10q26-qter and trisomy 11q13-qter | - | [150] |
| Combined 17p13.3 microdeletion with partial monosomy 21q21.3 |  | -^**^ | t(17;21)(p13.3;q21.2) | Mat | - | 1/1 | FISH and aCGH | Mild mental delay, dysmorphic features, and azoospermia | Associated with 17p microdeletion and partial monosomy 21q | - | [151] |
| Diffuse large B-cell lymphoma |  | -^**^ | t(14;18) | - | Median: 56y/ 16M, 10F | 61/86 | PCR | - | Associated with well-characterized Simian virus 40 status | - | [152] |
| APL |  | Rec^**^ | t(12;15;17)(q24;q24;q11) | - | 58y/M | 1/1 | RT-PCR and FISH | APL (FAB-M4) | Complex | - | [153] |
| Chronic myelomonocytic leukemia with monosomy 7 |  | -^**^ | t(15;17)(q22;q21) | - | 41y/M | 1/1 | Kar and RT-PCR | Secondary chronic myelomonocytic leukemia with monosomy 7 after treatment of APL | Associated with del(10)(q24) | - | [154] |
| ALL |  | -^**^  -^**^  -^**^  Rec^**^ | t(1;19)(q23;p13)  t(12;21)(p13;q22)  t(8;14)(q24;q32)  t(9;22)(q34;q11) | *De novo* | - | 1/298  10/298  2/298  20/298 | RHD banding and FISH | B cell-type ALL  Mature B-lineage phenotype and FAB L3 morphology B-ALL  Pediatric and adult ALL | Associated with other chromosomal translocations. | - | [155] |
| B-ALL |  | -^**^  -^**^  -^**^  -^**^ | t(1;19)(q23;p13)  t(12;21)(p13;q22)  t(4;11)(q21;q23)  t(8;14)(q24;q32) | - | Men: 7.4y/ 25M, 16F | 1/41  16/41  2/41  1/41 | RHG banding and FISH | - | Primary in 11 patients and associated with other chromosomal translocations in 5 patients. | - | [156] |
| XX maleness |  | -^**^ | Translocation of SRY to Xp terminal part in XX male | *De novo* | 32y/M | 1/1 | RHG banding and FISH | Azoospermia, normal sexual development and bilateral small testes | Primary | - | [157] |
| Infertility |  | Rob^**^  Rec^**^  Rec^**^  Rec^**^  Rec^*^ | t(13;14)(q10;q10)  t(3;7)(p24;p15)  t(4;10)(q34;q10)  t(7;20)(p12;p13)  t(9;13)(q33;q22) | - | Mean: 36.8y/M | 2/6  4/6  2/6  2/6  2/6 | FISH | Reproductive failure (recurrent miscarriage, infertility problem) | Primary | - | [158] |
| Mental retardation and spina bifida |  | Rec^*^ | t(2;3)(q35;p26.2) | *De novo* | 6y/F | 1/1 | R-banding and FISH | Mental retardation, mild growth, congenital malformation, and facial anomalies | Associated with partial trisomy 2q35 and partial monosomy 3p26 | No | [159] |
| CML with variant Ph-rearrangements |  | Rec^*^  Rec^*^  Rec^**^  Rec^**^  Rec^**^  Rec^**^  Rec^**^  Rec^**^  Rec^*^  Rec^*^  Rec^*^  Rec^**^  Rec^**^  Rec^**^  Rec^*^  Rec^**^  Rec^**^  Rec^**^  Rec^**^  Rec^**^  Rec^**^  Rec^**^  Rec^**^ | t(1;1;2;9;12;13;22)(q24;q31;p21;q34;q11.2)  t(1;1;9;22)(p34;q42;q34;q11.2)  t(1;9;22)(p35;q34;q11.2)  t(1;9;22)(p36;q34;q11.2)  t(10;9;22)(q25;q34;q11.2)  t(11;9;22)(q12;q34;q11.2)  t(3;9;22)(p14;q34;q11.2)  t(3;9;22)(q26;q34;q11.2)  t(4;9;22)(q13;q34;q11.2)  t(4;9;22)(q27;q34;q11.2)  t(4;9;22)(q34;q34;q11.2)  t(6;9;22)(q21;q34;q11.2)  t(6;9;22)(q22;q34;q11.2)  t(9;12;12;22)(q34;q21;p12;q11.2)  t(9;12;22)(q34;p13;q11.2)  t(9;13;22)(q34;q13;q11.2)  t(9;13;22)(q34;q31;q11.2)  t(9;17;22)(q34;q22;q11.2)  t(9;17;22)(q34;q23;q11.2)  t(9;19;22)(q34;q13;q11.2)  t(9;21;22)(q34;q22;q11.2)  t(9;7;22)(q34;p21;q11.2)  t(X;9;22)(p22;q34;q11.2) | - | - | 25/336 | R-banding and FISH | Similar to CML clinical features | Associated with deletions | - | [160] |
| RPL |  | -^†**^  -^‡**^ | t(3;4)(q28-,p16+;p16-,q28+)  t(3;4)(q28;p16) | - | -/F | 1/42  3/- | R banding and FISH | Recurrent miscarriage with congenital anomalies. Couples with normal endocrine function, normal ovarian function, and normal genital organs | Primary | - | [161] |
| Autism |  | -^†**^ | t(7;16)(p22.1;p11.2) | *De novo* | 10y/M | 1/1 | G banding, FISH, and RT-PCR | Inability to maintain eye contact, no recognized malformations, and severe language impairment | Primary | No | [162-164] |
| AML |  | -^*^ | t(X;10)(p10;p10) | - | 86y/M  27y/M | 2/- | Cytogenetics | AML with poor prognosis due to systemic candidiasis and relapse | Primary  Associated with other abnormalities | - | [165] |
| RPL |  | Rec^**^  Rec^**^  Rec^**^ | t(1;2)(p35;q33)  t(2;3)(p24;q26)  t(5;6)(q13;q24) | - | Mean: 33y/M | 3/67 | RHG banding | RPL due to male factor involvement with two or more failed pregnancies, oligo and asthenoteratospermia | Primary | - | [166] |
| Premature ovarian failure |  | Rec^**^ | t(12;19)(q13;q13) | - | -/F | - | FISH | Cessation of ovarian function before the age of 40, associated with elevated gonadotrophin serum levels | - | - | [167] |
| Synovial sarcoma |  | Rec^**^ | t(X;18)(p11.2;q11.2) | - | 18-27y/ M | 2/2 | Cytogenetic | Malignant mesenchymal tumor | - | - | [168] |
| Unbalanced translocation | UAE | Rec^‡**^ | t(2;11)(q37.3;q23.3) | Pat | 7y/M  2y/F | 2/2 | Kar | Normal phenotype but carriers of an unbalanced translocation | Primary | No | [169] |
| Down syndrome |  | -^**^ | t(14;21) | - | /M | 1/141 | Kar | Down syndrome | - | - | [170] |

**Abbreviations:** Rec: reciprocal, Rob: Robertsonian, y: years, m: months, d: days, fam: familial, pat: paternal, mat: maternal, ALL: acute lymphoblastic leukemia, AML: acute lymphocytic leukemia, RPL: recurrent pregnancy loss, CML: chronic myeloid leukemia, NHL: Non-Hodgkin lymphoma, MCL: Mantle cell lymphoma, CLL: Chronic lymphocytic leukemia, CHARGE syndrome: coloboma, heart defects, atresia choanae, growth retardation, genital abnormalities, and ear abnormalities, ADHD: attention deficit hyperactivity disorder, APL: Acute promyelocytic leukemia, Kar: karyotyping, FISH: fluorescence in situ hybridization, dc-FISH: dual color-FISH, mc-FISH: muliti-color-FISH, PCR: polymerase chain reaction, SNP array: single nucleotide polymorphisms array, WB: western blotting, NB: northern blotting, SB: southern blotting, IHC: immunohistochemistry, aMCB: array-proven multicolor banding, aCGH: array comparative genomic hybridization, CISS: chromosomal in situ suppression hybridization, CSF: cerebrospinal fluid, RT-qPCR: reverse transcriptase quantitative- polymerase chain reaction, MCB: multicolor banding, G banding: Giemsa banding, R banding: reverse banding, RHG banding: R-banding produced by heat denaturation followed by Giemsa staining, NA: not applicable.

^*^Unique translocation, ^**^Shared translocation, ^†^Balanced translocation, ^‡^Unbalanced translocation.

**References**

1. Zerrouki, R., et al., *The complex translocation (9; 14; 14) involving IGH and CEBPE genes suggests a new subgroup in B-lineage acute lymphoblastic leukemia.* Genetics and molecular biology, 2016. **39**: p. 7-13.

2. Mark-Vendel, E., et al., *Chromosomal translocations in Algerian Burkitt's lymphoma.* Lancet (London, England), 1983. **2**(8353): p. 788-788.

3. Philip, T., et al., *EBV‐positive Burkitt's lymphoma from algeria, with a three‐way rearrangement involving chromosomes 2, 8 and 9.* International journal of cancer, 1981. **28**(4): p. 417-420.

4. Belmokhtar, F., R. Belmokhtar, and A. Kerfouf, *Cytogenetic study of down syndrome in Algeria: Report and review.* Journal of Medical Sciences, 2016. **36**(2): p. 46.

5. Gaboon, N.E., et al., *Structural chromosomal abnormalities in couples with recurrent abortion in Egypt.* Turk J Med Sci, 2015. **45**(1): p. 208-13.

6. Nazmy, N., et al., *Familial reciprocal non robertsonian translocation t (14; 22) resulting in 22q11. 2 deletion syndrome.* The Turkish journal of pediatrics, 2019. **61**(5): p. 780-785.

7. Elhady, G.M., S. Kholeif, and N. Nazmy, *Chromosomal Aberrations in 224 Couples with Recurrent Pregnancy Loss.* Journal of Human Reproductive Sciences, 2020. **13**(4): p. 340.

8. Kumar, N., et al., *Primary tracheal synovial sarcoma: a rare clinical entity with diagnostic challenges.* Journal of the Egyptian National Cancer Institute, 2020. **32**(1): p. 1-6.

9. Hussen, D.F., et al., *Phenotypic and Molecular Cytogenetic Analysis of a Case of Monosomy 1p36 Syndrome due to Unbalanced Translocation.* Molecular Syndromology, 2020. **11**(5-6): p. 284-295.

10. El-Gilany, A.-H., et al., *Cytogenetic and comorbidity profile of Down syndrome in Mansoura University Children's Hospital, Egypt.* Indian journal of human genetics, 2011. **17**(3): p. 157-163.

11. Mekkawy, M.K., et al., *Cytogenetic spectrum of ovotesticular difference of sex development (OT DSD) among a large cohort of DSD patients and literature review.* Sexual development, 2019. **13**(5-6): p. 221-227.

12. Mazen, I., et al., *Unique karyotype: mos 46, X, dic (X; Y)(p22. 33; p11. 32)/45, X/45, dic (X; Y)(p22. 33; p11. 32) In an Egyptian patient with ovotesticular disorder of sexual development.* Sexual Development, 2013. **7**(5): p. 235-243.

13. Ashaat, N. and A. Husseiny, *Correlation between missed abortion and insertional translocation involving chromosomes 1 and 7.* Iranian journal of reproductive medicine, 2012. **10**(1): p. 15.

14. Mokhtar, M., et al., *Cytogenetic profile of Down syndrome in Alexandria, Egypt.* EMHJ-Eastern Mediterranean Health Journal, 9 (1-2), 37-44, 2003, 2003.

15. Mekkawy, M.K., et al., *An infertile azoospermic male with 45, X karyotype and a unique complex (Y; 14);(Y; 22) translocation: cytogenetic and molecular characterization.* Journal of assisted reproduction and genetics, 2018. **35**(8): p. 1503-1508.

16. Mikhail, F.M., et al., *A new translocation that rearranges the AML1 gene in a patient with T-cell acute lymphoblastic leukemia.* Cancer genetics and cytogenetics, 2002. **135**(1): p. 96-100.

17. Temtamy, S., et al., *COFS syndrome with familial 1; 16 translocation.* Clinical genetics, 1996. **50**(4): p. 240-243.

18. El-Ghammaz, A.M. and E. Abdelwahed, *Bortezomib-based induction improves progression-free survival of myeloma patients harboring 17p deletion and/or t (4; 14) and overcomes their adverse prognosis.* Annals of hematology, 2016. **95**(8): p. 1315-1321.

19. Atfy, M., N.M. Al Azizi, and A.M. Elnaggar, *Incidence of Philadelphia-chromosome in acute myelogenous leukemia and biphenotypic acute leukemia patients: and its role in their outcome.* Leukemia research, 2011. **35**(10): p. 1339-1344.

20. Mosad, E., M. Abdou, and A.H. Zaky, *Rearrangement of the myeloid/lymphoid leukemia gene in therapy-related myelodysplastic syndrome in patients previously treated with agents targeting DNA topoisomerase II.* Oncology, 2012. **83**(3): p. 128-134.

21. Zaki, M., et al., *Identification of a novel recessive RELN mutation using a homozygous balanced reciprocal translocation.* American Journal of Medical Genetics Part A, 2007. **143**(9): p. 939-944.

22. Mikhail, F.M., et al., *A novel gene, FGA7, is fused to RUNX1/AML1 in at (4; 21)(q28; q22) in a patient with T‐cell acute lymphoblastic leukemia.* Genes, Chromosomes and Cancer, 2004. **39**(2): p. 110-118.

23. Abd El-hameed, A., *De novo nodal diffuse large B-cell lymphoma: identification of biologic prognostic factors.* J Egypt Natl Canc Inst, 2005. **17**(1): p. 20-8.

24. Abdel-Ghaffar, H., et al., *Prognostic value of the t (14; 18)(q32; q21) in patients with diffuse large B-cell lymphoma.* Cancer investigation, 2010. **28**(4): p. 376-380.

25. Deghiedy, H., et al., *Diagnostic and prognostic utility of t (14; 18) in follicular lymphoma.* Acta Haematologica, 2007. **118**(4): p. 231-236.

26. Sorour, A. and D. Nafea, *Dual color FISH on CBF primary acute myeloid leukemia.* EGYPTIAN JOURNAL OF IMMUNOLOGY, 2008. **15**(2): p. 25-31.

27. El-Ruby, M., N. Hemly, and M.S. Zaki, *Maternal balanced translocation (4; 21) leading to an offspring with partial duplication of 4q and 21q without phenotypic manifestations of Down syndrome.* Genetic counseling, 2007. **18**(2): p. 217.

28. Abbas, O.M., N.A. Omar, and Z.K. Hassan, *T (14; 18) is not associated with mixed cryoglobulinemia or with clonal B cell expansion in egyptian patients with hepatitis C virus infection.* Journal of the Egyptian National Cancer Institute, 2008. **20**(2): p. 149-157.

29. Hagag, A.A., et al., *Prognostic impact of WT-1 gene expression in egyptian children with acute lymphoblastic leukemia.* Mediterranean journal of hematology and infectious diseases, 2016. **8**(1).

30. Abdalla, E.M., S.F. Kholeif, and R.M. Elshaffie, *Homozygosity for a Robertsonian Translocation (13q; 14q) in an otherwise healthy 44, xy man with a history of repeated fetal losses.* Laboratory Medicine, 2013. **44**(3): p. 254-257.

31. Mikhail, F., et al., *AML1 gene over-expression in childhood acute lymphoblastic leukemia.* Leukemia, 2002. **16**(4): p. 658-668.

32. El-Rifai, W.e., et al., *Follow-up of residual disease using metaphase-FISH in patients with acute lymphoblastic leukemia in remission.* Leukemia, 1997. **11**(5): p. 633-638.

33. El-Rifai, W.e., et al., *Chromosomal breakpoints and changes in DNA copy number in refractory acute myeloid leukemia.* Leukemia, 1997. **11**(7): p. 958-963.

34. Mohammed, Y.A., et al., *Chromosomal study in newborn infants with congenital anomalies in Assiut University hospital: Cross-sectional study.* Egyptian Journal of Medical Human Genetics, 2011. **12**(1): p. 79-90.

35. El-Dahtory, F.A.M., *Chromosomal abnormalities as a cause of recurrent abortions in Egypt.* Indian journal of human genetics, 2011. **17**(2): p. 82.

36. AbouEl-Ella, S.S., et al., *Study of congenital malformations in infants and children in Menoufia governorate, Egypt.* Egyptian Journal of Medical Human Genetics, 2018. **19**(4): p. 359-365.

37. Shawky, R.M. and R. Gamal, *C syndrome with skeletal anomalies, mental retardation, eyelid chalazion, Bitot’s spots and agenesis of the corpus callosum in an Egyptian child.* Egyptian Journal of Medical Human Genetics, 2017. **18**(1): p. 93-97.

38. Kassem, N., et al., *Novel mutations of the nucleophosmin (NPM-1) gene in Egyptian patients with acute myeloid leukemia: a pilot study.* J Egypt Natl Canc Inst, 2011. **23**(2): p. 73-8.

39. Kassem, N., et al., *CCAAT/enhancer binding protein α gene expression in Egyptian patients with acute myeloid leukemia.* Journal of the Egyptian National Cancer Institute, 2013. **25**(3): p. 115-120.

40. Abaza, H.M.H., et al., *Detection of 14q32 rearrangements in multiple myeloma, using simultaneous FISH analysis combined with immunofluorescence.* Hematology/Oncology and Stem Cell Therapy, 2015. **8**(2): p. 56-63.

41. Qaqish, B.a.M., et al., *Occupational exposure to pesticides and occurrence of the chromosomal translocation t(14;18) among farmers in Jordan.* Toxicology Reports, 2016. **3**: p. 225-229.

42. Kamal, N.R., C.A. Hanson, and G.W. Dewald, *Acute promyelocytic leukemia with t (15; 16; 17; 19) and unusual fluorescence in situ hybridization pattern with PML and RARA probes.* Cancer genetics and cytogenetics, 1996. **92**(1): p. 54-57.

43. Ismail, S., N. Ababneh, and A. Awidi, *Identification of atypical PML-RARA breakpoint in a patient with acute promyelocytic leukemia.* Acta haematologica, 2007. **118**(3): p. 183-187.

44. Ayesh, M., et al., *Cytogenetic and morphological analysis of de novo acute myeloid leukemia in adults: A single center study in Jordan.* Balkan Journal of Medical Genetics, 2012. **15**(1): p. 5-10.

45. Mustafa Ali, M.K., M.M. Sabha, and K.H. Al-Rabi, *Spontaneous subdural hematoma in a patient with Philadelphia chromosome-positive acute lymphoblastic leukemia with normal platelet count after dasatinib treatment.* Platelets, 2015. **26**(5): p. 491-494.

46. Ismail, S.I., et al., *Frequency of t (14; 18) in follicular lymphoma patients: geographical or technical variation.* International journal of laboratory hematology, 2009. **31**(5): p. 535-543.

47. Masri, A., et al., *Microarray delineation of familial chromosomal imbalance with deletion 5q35 and duplication 10q25 in a child showing multiple anomalies and dysmorphism.* American Journal of Medical Genetics Part A, 2014. **164**(5): p. 1254-1261.

48. Ellati, R.T., et al., *Clinicopathologic features of translocation renal cell carcinoma.* Clinical genitourinary cancer, 2017. **15**(1): p. 112-116.

49. Bara’a, M.Q., et al., *Occupational exposure to pesticides and occurrence of the chromosomal translocation t (14; 18) among farmers in Jordan.* Toxicology reports, 2016. **3**: p. 225-229.

50. Chebly, A., et al., *A rare case of acute myeloid leukemia with t (12; 19)(q13; q13).* Leukemia Research Reports, 2020. **14**: p. 100216.

51. Daniel, F., et al., *A single mass forming colonic primary mantle cell lymphoma.* Case reports in gastrointestinal medicine, 2016. **2016**.

52. Assaf, N., et al., *Molecular profiling of adult acute myeloid and lymphoid leukemia in a major referral center in Lebanon: a 10-year experience report and review of the literature.* Molecular biology reports, 2019. **46**(2): p. 2003-2011.

53. Rassy, E.E., et al., *Untreated chronic lymphocytic leukemia in Lebanese patients: an observational study using standard karyotyping and FISH.* International journal of hematologic oncology, 2017. **6**(4): p. 105-111.

54. Millot, F., et al., *Additional cytogenetic abnormalities and variant t (9; 22) at the diagnosis of childhood chronic myeloid leukemia: The experience of the I nternational R egistry for C hronic M yeloid L eukemia in C hildren and A dolescents.* Cancer, 2017. **123**(18): p. 3609-3616.

55. Mahfouz, R., et al., *Molecular frequency of BCL2/JH t (14; 18) using PCR among Lebanese patients with follicular lymphoma: another piece of the geographical map revealed.* Molecular biology reports, 2007. **34**(4): p. 271-274.

56. Farra, C., et al., *Complex translocation (8; 12; 21): a new variant of t (8; 21) in acute myeloid leukemia.* Cancer genetics and cytogenetics, 2004. **155**(2): p. 138-142.

57. Zahed, L., et al., *Ring chromosome 18q and jumping translocation 18p in an adult male with hypergonadotrophic hypogonadism.* American Journal of Medical Genetics Part A, 2004. **129**(1): p. 25-28.

58. Farah, R.A., et al., *A multicenter experience from Lebanon in childhood and adolescent acute myeloid leukemia: high rate of early death in childhood acute promyelocytic leukemia.* Mediterranean journal of hematology and infectious diseases, 2015. **7**(1).

59. Haddad, F.G., et al., *An unusual case of chronic lymphocytic leukemia with trisomy 12 and t (14; 18) and a favorable response to ibrutinib.* Leukemia Research Reports, 2021. **15**: p. 100245.

60. Diejomaoh, M.F., et al., *Consecutive successful pregnancies subsequent to intravenous immunoglobulin therapy in a patient with recurrent spontaneous miscarriage.* International medical case reports journal, 2015. **8**: p. 337.

61. Zámečníkova, A., S. Al Bahar, and P. Ramesh, *Simultaneous occurrence of t (9; 22)(q34; q11. 2) and t (16; 16)(p13; q22) in a patient with chronic myeloid leukemia in blastic phase.* Cancer genetics and cytogenetics, 2008. **183**(2): p. 109-113.

62. Zámecˇníkova, A., S. Al Bahar, and R. Pandita, *Unusual location of BCR-ABL1 fusion sequences in a chronic myeloid leukemia patient.* Hematology, 2012. **17**(6): p. 321-324.

63. Adriana, Z. and S. Al Bahar, *Novel four-way Ph translocation t (9; 22; 7; 1)(q34; q11; q22; p13) in a chronic myeloid leukemia patient receiving tyrosine kinase inhibitor therapy.* International journal of hematology, 2012. **95**(3): p. 315-319.

64. Al-Bahar, S., A. Zámečníkova, and R. Pandita, *Frequency and type of chromosomal abnormalities in childhood acute lymphoblastic leukemia patients in Kuwait: a six-year retrospective study.* Medical Principles and Practice, 2010. **19**(3): p. 176-181.

65. Zafer, E., et al., *Autosomal dominant congenital cataract in a Libyan Jewish family: cosegregation with a reciprocal chromosomal translocation [t (3; 5)(p22. 3; p15. 1)].* Molecular vision, 2008. **14**: p. 530.

66. El Andaloussi, A. and C. Bilhou-Nabera, *New complex chromosomal translocation in chronic myeloid leukaemia: T (9; 18; 22)(q34; p11; q11).* Journal of Biomedicine and Biotechnology, 2007. **2007**.

67. Belkady, B., et al., *Chromosomal abnormalities in patients with intellectual disability: a 21-year retrospective study.* Human heredity, 2018. **83**(5): p. 274-282.

68. Elkarhat, Z., et al., *Chromosomal abnormalities in couples with recurrent spontaneous miscarriage: a 21-year retrospective study, a report of a novel insertion, and a literature review.* Journal of assisted reproduction and genetics, 2019. **36**(3): p. 499-507.

69. Chebihi, Z.T., et al., *The rare translocation t (14; 21)(q11; q22) detected in a Moroccan patient with T-cell acute lymphoblastic leukemia.* Leukemia research reports, 2019. **11**: p. 1-4.

70. Hamdaoui, H., et al., *Cytogenetic and FISH analysis of 93 multiple myeloma Moroccan patients.* Molecular genetics & genomic medicine, 2020. **8**(9): p. e1363.

71. Latrech, H., H. Madar, and A. Gaouzi, *Combination of gonadal dysgenesis and monosomy X with a Novo translocation (13, 14).* Case reports in endocrinology, 2018. **2018**.

72. Rebahi, H., M. Ait Sliman, and A.-R. El Adib, *Chronic myeloid leukemia and cesarean section: the anesthesiologist’s point of view.* Case reports in obstetrics and gynecology, 2018. **2018**.

73. Amasdl, S., et al., *Familial X/Y Translocation Encompassing ARSE in Two Moroccan Siblings with Sensorineural Deafness.* Cytogenetic and genome research, 2017. **153**(2): p. 66-72.

74. Jaouad, I., et al., *Cytogenetic and epidemiological profiles of Down syndrome in a Moroccan population: a report of 852 cases.* Singapore medical journal, 2010. **51**(2): p. 133.

75. Bennour, A., et al., *Translocation t (X; 10)(p10; p10): a rare chromosomal abnormality in a new born female with acute myeloid leukemia.* Medical Oncology, 2012. **29**(2): p. 1134-1136.

76. Hamdaoui, H., et al., *Near tetrapoloid karyotype with translocation t (11; 14) in a Moroccan patient with amyloid light-chain amyloidosis and multiple myeloma.* Leukemia Research Reports, 2020. **14**: p. 100217.

77. Quessar, A., et al., *High Incidence and Curability of Patients with Acute Myeloblastic Leukemia (AML) and t (8; 21) Treated On a Uniform Protocol in Casablanca, Morocco*. 2009, American Society of Hematology.

78. Udayakumar, A.M., et al., *Complex t (8; 13; 21)(q22; q14; q22)–A Novel Variant of t (8; 21) in a Patient with Acute Myeloid Leukemia (AML–M2).* Archives of medical research, 2008. **39**(2): p. 252-256.

79. Farra, C., et al., *Complex translocation (8;12;21): a new variant of t(8;21) in acute myeloid leukemia.* Cancer Genet Cytogenet, 2004. **155**(2): p. 138-42.

80. Goud, T.M., et al., *Cytogenetic studies in couples with recurrent miscarriage in the Sultanate of Oman.* Reproductive biomedicine online, 2009. **18**(3): p. 424-429.

81. Goud, T.M., et al., *Importance of FISH combined with Morphology, Immunophenotype and Cytogenetic Analysis of Childhood/ Adult Acute Lymphoblastic Leukemia in Omani Patients.* Asian Pac J Cancer Prev, 2015. **16**(16): p. 7343-50.

82. Abuhamda, A., A. Elsous, and F.A. Sharif, *Partial Trisomy of Chromosome 13 with a Novel Translocation (8; 13) and Unique Clinical Presentation in a Palestinian Infant.* Case reports in medicine, 2019. **2019**.

83. Ibrahim, F., et al., *Clinico-pathological profile of acute promyelocytic leukaemia at Al-Amal Oncology-Haematology Centre, Qatar.* EMHJ-Eastern Mediterranean Health Journal, 16 (9), 958-965, 2010, 2010.

84. Soliman, D.S., et al., *Chronic Myeloid Leukemia with cryptic Philadelphia translocation and extramedullary B-lymphoid blast phase as an initial presentation.* Acta Bio Medica: Atenei Parmensis, 2018. **89**(Suppl 3): p. 38.

85. Mostafavipour, S., et al., *Chromosomal abnormalities in childhood acute nonlymphocytic leukemia (M4).* Cancer genetics and cytogenetics, 1991. **57**(2): p. 195-200.

86. Al‐Mulla, N.A., et al., *Childhood acute lymphoblastic leukemia in the Middle East and neighboring countries: A prospective multi‐institutional international collaborative study (CALLME1) by the Middle East Childhood Cancer Alliance (MECCA).* Pediatric blood & cancer, 2014. **61**(8): p. 1403-1410.

87. Ali, E.A.H., S. Al-Akiki, and M.A. Yassin, *A Case Report of BCR-ABL-JAK2-Positive Chronic Myeloid Leukemia with Complete Hematological and Major Molecular Response to Dasatinib.* Case Reports in Oncology, 2021. **14**(1): p. 690-694.

88. Arafa, M.M., et al., *Chromosomal abnormalities in infertile men with azoospermia and severe oligozoospermia in Qatar and their association with sperm retrieval intracytoplasmic sperm injection outcomes.* Arab journal of urology, 2018. **16**(1): p. 132-139.

89. Elsayed, A.K., et al., *Generation of a human induced pluripotent stem cell line (QBRIi009-A) from a patient with a heterozygous deletion of FOXA2.* Stem cell research, 2020. **42**: p. 101705.

90. Turki, R.F., et al., *Associations of recurrent miscarriages with chromosomal abnormalities, thrombophilia allelic polymorphisms and/or consanguinity in Saudi Arabia.* BMC medical genetics, 2016. **17**(1): p. 15-23.

91. Ahmed, A.M., et al., *Improved Outcomes of Childhood Acute Lymphoblastic Leukemia: A Retrospective Single Center Study in Saudi Arabia.* Asian Pacific journal of cancer prevention: APJCP, 2019. **20**(11): p. 3391.

92. Al-Maghrabi, J., S. Mufti, and W. Gomaa, *The incidence of renal cell carcinoma associated with Xp11. 2 translocation/TFE3 gene fusion in Saudi adult patients with renal cancer: a retrospective tissue microarray analysis.* Polish Journal of Pathology, 2019. **69**(4): p. 376-383.

93. AlMajhad, N.A., et al., *Neurological expression of an inherited translocation of chromosomal 1 and 7.* Neurosciences Journal, 2017. **22**(1): p. 62-64.

94. Almesned, R.K., et al., *Male Infertility in Robertsonian Translocation: A Case Report.* The American journal of case reports, 2020. **21**: p. e921616-1.

95. Cherian, M.P., *Influence of HLA DQ 2/8 genotypes in predisposing type 1 diabetes in siblings of a Saudi family with paternally inherited chromosomal translocations.* Journal of Pediatric Endocrinology and Metabolism, 2012. **25**(5-6): p. 569-572.

96. Saleh, D., et al., *Desmoplastic Small Round Cell Tumor of Pancreatic Origin in a Young Child: A Case Report and Review of Literature.* The American Journal of Case Reports, 2020. **21**: p. e922762-1.

97. Abu-Zaid, A., et al., *Desmoplastic small round cell tumor of stomach.* Case reports in gastrointestinal medicine, 2013. **2013**.

98. Aljabry, M., *Complex karyotype with novel translocation in pure erythroid leukemia patient.* Hematology reports, 2015. **7**(1).

99. Elyamany, G., et al., *De Novo CD5 negative blastic mantle cell lymphoma presented with massive bone marrow necrosis without adenopathy or organomegaly.* Case reports in hematology, 2015. **2015**.

100. Buzaid, A.N. and A.M. Al-Amri, *Sudden visual loss as an initial manifestation of chronic myeloid leukemia.* Saudi journal of medicine & medical sciences, 2017. **5**(3): p. 278.

101. Iqbal, M.A., et al., *A case of presumptive monosomy 21 re‐diagnosed as unbalanced t (5p; 21q) by FISH and review of literature.* American journal of medical genetics, 1997. **70**(2): p. 174-178.

102. Khalifa, O.A., et al., *Terminal 4q deletion and 8q duplication in a patient with CHARGE-like features.* European journal of medical genetics, 2011. **54**(2): p. 173-176.

103. Dewan, M., T.S. Malatani, and M.A. Ansari, *Lessons to be learned: a case study approach: Malignant melanoma of soft tissue.* The journal of the Royal Society for the Promotion of Health, 2005. **125**(1): p. 42-46.

104. Bhat, J.N., et al., *Severe hypercalcemia in an infant with unbalanced translocation of chromosomes 2 and 8: a possible contribution of 2p duplication.* Journal of Pediatric Endocrinology and Metabolism, 2021. **34**(5): p. 659-666.

105. Niazi, M., P. Van Dijken, and K. Al Moutaery, *A patient with meningioma showing multiple cytogenetic abnormalities and a constitutional translocation (3; 9)(q13. 3; q22).* Cancer genetics and cytogenetics, 1998. **105**(1): p. 11-13.

106. Nasrin, N., et al., *Apparent absence of bcl-2 rearrangement in ten Saudi follicular lymphomas: a preliminary report.* Annals of Saudi medicine, 1994. **14**(3): p. 219-224.

107. Alhashem, A.M., et al., *Intellectual Disability in Two Brothers Caused by De Novo Novel Unbalanced Translocation (13; 18)(q34, q23) and De Novo Microdeletion 6q25 Syndrome.* Cureus, 2020. **12**(1).

108. Alghasham, N., et al., *Plasma cell leukemia: Clinicopathologic, immunophenotypic and cytogenetic characteristics of 4 cases.* Hematol Oncol Stem Cell Ther, 2015. **8**(2): p. 71-7.

109. Murthy, S.S., et al., *Ewing sarcoma with emphasis on extra-skeletal Ewing sarcoma: a decade’s experience from a single centre in India.* Clinical Pathology, 2020. **13**: p. 2632010X20970210.

110. Alowaysi, M., et al., *Generation of two iPSC lines (KAUSTi001-A, KAUSTi002-A) from a rare high-grade Klinefelter Syndrome patient (49-XXXXY) carrying a balanced translocation t (4, 11)(q35, q23).* Stem Cell Research, 2020. **49**: p. 102098.

111. Colak, D., et al., *Genomic and transcriptomic analyses distinguish classic Rett and Rett-like syndrome and reveals shared altered pathways.* Genomics, 2011. **97**(1): p. 19-28.

112. Al-Balbeesi, A.O., *Atrophic dermatofibrosarcoma protuberans and enlargement with pregnancy: Case report and literature review.* Journal of the Saudi Society of Dermatology & Dermatologic Surgery, 2012. **16**(1): p. 21-23.

113. Owaidah, T.M., F.I. Rawas, and N.B. Elkum, *Expression of CD66c and CD25 in acute lymphoblastic leukemia as a predictor of the presence of BCR/ABL rearrangement.* Hematology/oncology and stem cell therapy, 2008. **1**(1): p. 34-37.

114. Umiel, T., et al., *Undifferentiated leukemia of infancy with t (11: 17) chromosomal rearrangement coexpressing myeloid and B cell restricted antigens.* Cancer, 1987. **59**(6): p. 1143-1149.

115. Muddathir, A.R.M., et al., *Distribution of fusion transcripts and its clinical impact in patients with acute myeloid leukemia in Sudan.* International Journal of Health Sciences, 2021. **15**(2): p. 21.

116. Wafa, A., et al., *Childhood pre-B cell acute lymphoblastic leukemia with translocation t (1; 19)(q21. 1; p13. 3) and two additional chromosomal aberrations involving chromosomes 1, 6, and 13: a case report.* Journal of medical case reports, 2017. **11**(1): p. 1-6.

117. Alhalabi, N., et al., *De novo Balanced Robertsonian Translocation rob (22; 22)(q10; q10) in a Woman with Recurrent Pregnancy Loss: A Rare Case.* Journal of reproduction & infertility, 2018. **19**(1): p. 61.

118. Wafa, A., et al., *Acute promyelocytic leukemia with the translocation t (15; 17)(q22; q21) associated with t (1; 2)(q42~ 43; q11. 2~ 12): a case report.* Journal of medical case reports, 2016. **10**(1): p. 1-5.

119. Al-Achkar, W., A. Wafa, and T. Liehr, *A new t (9; 11; 20; 22)(q34; p11. 2; q11. 21; q11) in a Philadelphia-positive chronic myeloid leukemia case.* Oncology letters, 2013. **5**(2): p. 605-608.

120. Al‑Achkar, W., et al., *Three‑way Philadelphia translocation t (9; 10; 22)(q34; p11. 2; q11. 2) as a secondary abnormality in an imatinib mesylate‑resistant chronic myeloid leukemia patient.* Oncology letters, 2013. **5**(5): p. 1656-1658.

121. Al-Achkar, W., A. Wafa, and S. Almedani, *BCR translocation to derivative chromosome 2: A new case of chronic myeloid leukemia with a complex variant translocation and Philadelphia chromosome.* Oncology letters, 2010. **1**(3): p. 445-447.

122. Al Achkar, W., et al., *De novo acute myeloid leukemia subtype‑M4 with initial trisomy 8 and later acquired t (3; 12)(q26; p12) leading to ETV6/MDS1/EVI1 fusion transcript expression: A case report.* Oncology letters, 2014. **7**(3): p. 787-790.

123. Al‑Achkar, W., et al., *A novel cytogenetic abnormality t (7; 8)(p11. 2: q11. 2) and a four-way Philadelphia translocation in an imatinib mesylate‑resistant chronic myeloid leukemia patient.* Oncology letters, 2013. **5**(2): p. 617-620.

124. Moassass, F., et al., *Down syndrome associated childhood myeloid leukemia with yet unreported acquired chromosomal abnormalities and a new potential adverse marker: dup (1)(q25q44).* Molecular cytogenetics, 2018. **11**(1): p. 1-6.

125. Al-Achkar, W., et al., *A chronic myeloid leukemia case with a unique variant Philadelphia translocation: t (9; 22; 21)(q34; q11; p12).* Oncology letters, 2012. **3**(5): p. 1027-1029.

126. Al Achkar, W., et al., *A rare case of chronic myeloid leukemia with secondary chromosomal changes including partial trisomy 17q21 to 17qter and partial monosomy of 16p13. 3.* Molecular cytogenetics, 2010. **3**(1): p. 1-4.

127. Al‐Achkar, W., A. Wafa, and R.A. Jarjour, *A new case of de novo translocation (12; 17; 18)(q21. 2; q22; q21. 1) and cranio‐cerebello‐cardiac (3C) syndrome.* American Journal of Medical Genetics Part A, 2011. **155**(3): p. 648-651.

128. Al Achkar, W., et al., *A rare chronic myeloid leukemia case with Philadelphia chromosome, BCR-ABL e13a3 transcript and complex translocation involving four different chromosomes.* Oncology letters, 2010. **1**(5): p. 797-800.

129. Wafa, A., et al., *Masked inv dup (22)(q11. 23), tetrasomy 8 and trisomy 19 in a blast crisis-chronic myeloid leukemia after interrupted Imatinib-treatment.* Molecular cytogenetics, 2015. **8**(1): p. 1-5.

130. Al-Achkar, W., A. Wafa, and T. Liehr, *Complex translocation involving four chromosomes in a novel Philadelphia-positive chronic myeloid leukemia case.* Oncology letters, 2011. **2**(2): p. 273-276.

131. Al-Achkar, W., et al., *Hyperdiploidy associated with T315I mutation in BCR-ABL kinase domain in an accelerated phase-chronic myeloid leukemia case.* Molecular cytogenetics, 2014. **7**(1): p. 1-6.

132. Al-Achkar, W., A. Wafa, and M. Nweder, *A complex translocation t (5; 9; 22) in Philadelphia cells involving the short arm of chromosome 5 in a case of chronic myelogenous leukemia.* J Exp Clin Cancer Res, 2007. **26**(3): p. 411-5.

133. Walid, A.-A., et al., *An adult B-cell precursor acute lymphoblastic leukemia with multiple secondary cytogenetic aberrations.* Molecular cytogenetics, 2014. **7**(1): p. 1-5.

134. Wafa, A., et al., *A high complex karyotype involving eleven chromosomes including three novel chromosomal aberrations and monoallelic loss of TP53 in case of follicular lymphoma transformed into B-cell lymphoblastic leukemia.* Molecular cytogenetics, 2016. **9**(1): p. 1-7.

135. Al Achkar, W., et al., *Novel complex translocation involving 5 different chromosomes in a chronic myeloid leukemia with Philadelphia chromosome: a case report.* Mol Cytogenet, 2009. **2**: p. 21.

136. Al Achkar, W., et al., *A unique complex translocation involving six different chromosomes in a case of childhood acute lymphoblastic leukemia with the Philadelphia chromosome and adverse prognosis.* Oncology Letters, 2010. **1**(5): p. 801-804.

137. Al Achkar, W., et al., *A rare case of Philadelphia chromosome-positive chronic myelogenous leukemia with inversion in chromosome 9 and t (10; 17).* Oncology letters, 2010. **1**(5): p. 793-795.

138. Awartani, K.A. and M.S. Al Shabibi, *Description of cytogenetic abnormalities and the pregnancy outcomes of couples with recurrent pregnancy loss in a tertiary-care center in Saudi Arabia.* Saudi medical journal, 2018. **39**(3): p. 239.

139. Mahjoub, M., et al., *Chromosomal segregation in spermatozoa of five Robertsonian translocation carriers t (13; 14).* Journal of assisted reproduction and genetics, 2011. **28**(7): p. 607-613.

140. Ayed, W., et al., *Chromosomal abnormalities in 163 Tunisian couples with recurrent miscarriages.* Pan African Medical Journal, 2017. **28**(1): p. 158.

141. Amor, M.H., et al., *Neuronal migration genes and a familial translocation t (3; 17): candidate genes implicated in the phenotype.* BMC medical genetics, 2020. **21**(1): p. 1-11.

142. Bayou, N., et al., *De novo balanced translocation t (7; 16)(p22. 1; p11. 2) associated with autistic disorder.* Journal of Biomedicine and Biotechnology, 2008. **2008**.

143. Bennour, A., et al., *Molecular cytogenetic study of derivative chromosome 9 deletion in chronic myeloid leukemia patients.* Med Oncol, 2012. **29**(2): p. 1151-60.

144. Bennour, A., et al., *Molecular cytogenetic characterization of Philadelphia-negative rearrangements in chronic myeloid leukemia patients.* Journal of cancer research and clinical oncology, 2011. **137**(9): p. 1329-1336.

145. Bennour, A., et al., *Comprehensive analysis of BCR/ABL variants in chronic myeloid leukemia patients using multiplex RT-PCR.* Clinical laboratory, 2012. **58**(5): p. 433.

146. Gmidene, A., et al., *Molecular cytogenetic aberrations in Tunisian patients with multiple myeloma identified by cIg-FISH in fixed bone marrow cells.* Cytogenetic and genome research, 2012. **136**(1): p. 44-49.

147. Yamada, O., et al., *Emergence of a BCR-ABL translocation in a patient with the JAK2V617F mutation: evidence for secondary acquisition of BCR-ABL in the JAK2V617F clone.* Journal of clinical oncology: official journal of the American Society of Clinical Oncology, 2014. **32**(21): p. e76-e79.

148. Ben-Abdallah-Bouhjar, I., et al., *Chromosomal microarray analysis of functional Xq27-qter disomy and deletion 3p26. 3 in a boy with Prader–Willi like features and hypotonia.* European journal of medical genetics, 2012. **55**(8-9): p. 461-465.

149. Bennour, A., et al., *A novel t (3; 12)(q21; p13) translocation in a patient with accelerated chronic myeloid leukemia after imatinib and nilotinib therapy.* Cancer biology & medicine, 2013. **10**(1): p. 47.

150. Tinsa, F., et al., *Monosomy 10q26-qter and trisomy 11q13-qter as a result of de novo unbalanced translocation.* Journal of applied genetics, 2009. **50**(3): p. 289-291.

151. Hannachi, H., et al., *Clinical and molecular characterization of a combined 17p13. 3 microdeletion with partial monosomy 21q21. 3 in a 26-year-old man.* Cytogenetic and genome research, 2011. **135**(2): p. 102-110.

152. Amara, K., et al., *Presence of simian virus 40 in diffuse large B-cell lymphomas in Tunisia correlates with germinal center B-cell immunophenotype, t (14; 18) translocation, and P53 accumulation.* Modern Pathology, 2008. **21**(3): p. 282-296.

153. Bennour, A., et al., *A PML/RARA chimeric gene on chromosome 12 in a patient with acute promyelocytic leukemia (M4) associated with a new variant translocation: t (12; 15; 17)(q24; q24; q11).* Medical Oncology, 2013. **30**(1): p. 1-5.

154. Jeddi, R., et al., *Secondary chronic myelomonocytic leukemia with monosomy 7 after successful treatment of acute promyelocytic leukemia.* Pathologie Biologie, 2008. **56**(3): p. 162-163.

155. Gmidene, A., et al., *Cytogenetic analysis of 298 newly diagnosed cases of acute lymphoblastic leukaemia in Tunisia.* Hematological oncology, 2008. **26**(2): p. 91-97.

156. Gmidene, A., et al., *ETV6-RUNX1 rearrangement in Tunisian pediatric B-lineage acute lymphoblastic leukemia.* Advances in hematology, 2009. **2009**.

157. Abdelmoula, N.B., et al. *Skewed X-chromosome inactivation pattern in SRY positive XX maleness: a case report and review of literature*. in *Annales de genetique*. 2003. Elsevier.

158. Hajlaoui, A., et al., *Sperm fluorescent in situ hybridisation study of interchromosomal effect in six Tunisian carriers of reciprocal and Robertsonian translocations.* Andrologia, 2018. **50**(4): p. e12949.

159. Abdallah, I.B., et al., *Chromosomal microarray analysis in a girl with mental retardation and spina bifida.* Pediatric neurology, 2011. **44**(1): p. 65-68.

160. Bennour, A., et al., *Molecular cytogenetic characterization of variant Philadelphia translocations in chronic myeloid leukemia: genesis and deletion of derivative chromosome 9.* Cancer genetics and cytogenetics, 2009. **194**(1): p. 30-37.

161. Hajlaoui, A., et al., *Subtelomeric rearrangements in patients with recurrent miscarriage.* International journal of fertility & sterility, 2018. **12**(3): p. 218.

162. Bayou, N., et al., *The creatine transporter gene paralogous at 16p11.2 is expressed in human brain.* Comp Funct Genomics, 2008. **2008**: p. 609684.

163. Bayou, N., et al., *De novo balanced translocation t (7;16) (p22.1; p11.2) associated with autistic disorder.* Journal of biomedicine & biotechnology, 2008. **2008**: p. 231904-231904.

164. Bayou, N., et al., *Exploring the 7p22. 1 chromosome as a candidate region for autism.* Journal of biomedicine and biotechnology, 2010. **2010**.

165. Bennour, A., et al. *TRANSLOCATION (X; 10)(P10; P10): A RARE BUT NON RANDOM CHROMOSOMAL ABNORMALITY IN ACUTE MYELOID LEUKEMIA*. in *HAEMATOLOGICA-THE HEMATOLOGY JOURNAL*. 2010. FERRATA STORTI FOUNDATION VIA GIUSEPPE BELLI 4, 27100 PAVIA, ITALY.

166. Frikha, R., et al., *Assessment of male factor involved in recurrent pregnancy loss: A preliminary study.* Middle East Fertility Society Journal, 2018. **23**(3): p. 238-240.

167. Ayed, W., et al., *Cytogenetic abnormalities in Tunisian women with premature ovarian failure.* Comptes rendus biologies, 2014. **337**(12): p. 691-694.

168. Bellakhdhar, M., et al., *Laryngeal synovial sarcoma: Report of 2 cases.* Journal of the Egyptian National Cancer Institute, 2018. **30**(4): p. 173-176.

169. Al Gazali, L. and R. Quaife, *Two sibs with unbalanced translocations in the Waardenburg gene region.* Journal of medical genetics, 1993. **30**(7): p. 607.

170. Murthy, S.K., et al., *Incidence of Down syndrome in Dubai, UAE.* Medical Principles and Practice, 2007. **16**(1): p. 25-28.
